# The relationship between lipoprotein(a) and risk of cardiovascular disease: a Mendelian randomization analysis

**DOI:** 10.1186/s40001-022-00825-6

**Published:** 2022-10-27

**Authors:** Shiyue Wang, Li Zha, Jian Chen, Dongjie Du, Danyang Liu, Ming Zhong, Rongfang Shang, Dongxue Sun, Chang Sun, Enze Jin

**Affiliations:** The Fourth Affiliated Hospital of Harbin Medical University Cardiovascular Medical Department, Harbin, 150000 Heilongjiang China

**Keywords:** Lipoprotein(a), Mendelian randomization, Cardiovascular disease

## Abstract

**Background:**

Lipoprotein(a) [Lp(a)] is one of the residual risk factors for cardiovascular disease (CVD) in the setting of optimal low-density lipoprotein cholesterol (LDL-C). The association between Lp(a) and CVD is still in the exploratory phase, with few studies indicating a causal connection between Lp(a) and various CVD.

**Methods:**

Lp(a) (*n* = 377,590) was a genome-wide association study (GWAS) based on European populations from Neale Lab. Large GWAS datasets for CVD, including aortic aneurysm(AA) (*n* = 209,366), atrial fibrillation(AF) (*n* = 1,030,836), coronary heart disease(CHD) (*n* = 361,194), secondary hypertension(HBP) (*n* = 164,147), heart failure(HF) (*n* = 208,178), ischemic stroke (IS) (*n* = 218,792), large artery atherosclerosis stroke(ISL) (*n* = 150, 765), small vessel stroke(ISS) (*n* = 198,048), lacunar stroke(LIS) (*n* = 225,419), and pulmonary embolism(PE) (*n* = 218,413) were also based on European populations. We performed separate univariate two-sample Mendelian randomization (MR) analysis for Lp(a) and CVD as described above. We evaluated this connection mainly using the random-effects inverse variance weighted technique(IVW1) with a 95% confidence interval (CI) for the odds ratio (OR). This was supplemented by MR-Egger, weighted median, maximum likelihood, penalized weighted median, and fixed-effects inverse variance weighted methods. MR-PRESSO offers another means of statistical detection.

**Results:**

Our two-sample MR, which was predominately based on IVW1, revealed a causal relationship between Lp(a) and AA (OR = 1.005, 95%CI: 1.001–1.010, *P* = 0.009), CHD (OR = 1.003, 95%CI 1.001–1.004, *P* = 0.010), and ISL (OR = 1.003, 9 5%CI 1.002–1.004, *P* = 9.50E−11), in addition, there is no causal association with AF, HBP, HF, IS, ISS, LIS, or PE. Similar conclusions were reached by the MR-PRESSO method.

**Conclusion:**

This MR study suggested a causal relationship between Lp(a) and CHD, AA, and ISL, but not associated with AF, HF, IS, LIS, ISS, HBP, or PE. Our work further verifies the association between Lp(a) and various CVD, resulting in improved Lp(a) management and a reduction in the prevalence of CVD.

**Supplementary Information:**

The online version contains supplementary material available at 10.1186/s40001-022-00825-6.

## Introduction

Cardiovascular disease (CVD), including heart and peripheral vascular disease, is the leading cause of death in the United States and worldwide [1, 2], and the social health and economic burden of its high mortality and disability rates are increasing.

Lipoprotein(a) [Lp(a)] is formed by the covalent binding of apolipoprotein A to apolipoprotein B via disulfide bonds [3], whose concentration is inversely related to the size of apolipoprotein A and whose 90% level is determined genetically [4]. Lp(a) has risen to become a recognized risk factor for CVD, when low-density lipoprotein cholesterol (LDL-C) is 104 mg/dl, Lp(a) is more closely associated with CVD than LDL-C and is a residual risk factor for CVD [5]. Its main physiopathological mechanisms include atherogenesis, promotion of inflammation and thrombosis [6]. Unlike LDL-C, despite the fact that Lp(a) has been linked to CVD, definitive proof of a causal relationship remains to be proven. A large epidemiological study demonstrated that the risk ratio for coronary heart disease (CHD) after adjustment for age and sex was 1.16 (95% CI 1.11–1.22) [7], other epidemiological studies [8, 9] and meta-analyses [10] have confirmed that Lp(a) with the development of CHD was significant. The association between Lp(a) and other CVD is still in dispute. Lp(a) has been identified in observational studies as a risk factor for atrial thrombi in atrial fibrillation (AF) patients, there is no connection between Lp(a) and incident AF events [11]. However, it is limited by the number of AF cases. Lp(a) is an independent risk factor for ischemic stroke(IS) [12], but studies on Lp(a) and stroke type are scarce. We performed Mendelian randomization(MR) analysis of large artery atherosclerosis stroke(ISL), small vessel stroke(ISS), and lacunar stroke(LIS). The relationship between Lp(a) and heart failure(HF) is inconclusive, Lp(a) and HF were not associated in a community study, while another observational study reported an association [13]. Limited evidence suggests a link between Lp(a) and hypertension (HBP), evidence from clinical sources indicates that approximately 30% of hypertensive patients have elevated Lp(a) levels [14]. A study pointed out that Lp(a) plays an important and direct role in thrombosis and reinforcement of the aortic wall of aneurysms [15], however, the correlation between Lp(a) and aortic aneurysm (AA) was not elucidated. The role of Lp(a) in venous embolic events such as pulmonary embolism(PE) is controversial, potential pathogenic mechanisms of Lp(a) include its similarity to fibrinogen, leading to reduced fibrin synthesis and fibrinolysis inhibition, the tendency of Lp(a) to oxidize upon entry into the vessel wall, and the production of highly immunogenic and pro-inflammatory phospholipids [16, 17], and whether these effects play an important role in PE is unclear [18]. Notably, the relationship between Lp(a) and various CVD is controversial, observational studies are susceptible to confounding factors and reverse causality, and the corresponding conclusions can be biased. And few studies on the causal relationship between Lp(a) and various CVD.

Genetic variants are used as instrument variables (IVs) in MR analysis, a strong tool for determining the relationship between exposures and diseases [19]. The confounders of individuals being randomly allocated genetic variants at the moment of conception can be greatly reduced using MR analysis. Furthermore, the possibility of reverse causation is reduced because the presence of the disease has no effect on people’s genotypes [20]. In this study, we performed a univariate MR to explore whether genetic evidence for Lp(a)-related traits were significantly associated with CVD risks.

### Materials and methods

**Fig. 1:**
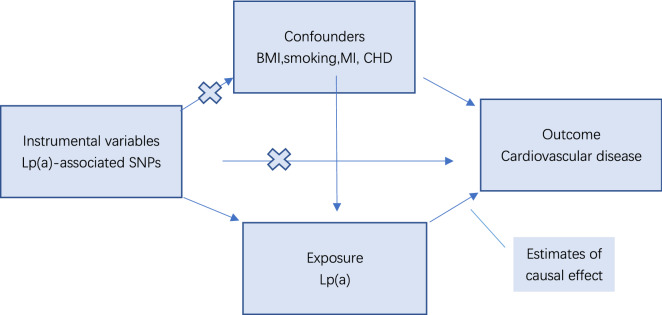
Schematic diagram of the MR assumptions underpinning an MR analysis of the association between Lipoprotein(a) levels and different CVD. *Lp(a)* lipoprotein(a), *BMI* body mass index, *MI* myocardial infarction, *CHD* coronary heart disease, *MR* Mendelian randomization

This is a univariate two-sample MR study with three major assumptions based on a publicly available GWAS. First, there must be a strong and independent relationship between the chosen instrumental variable, single nucleotide polymorphism, and Lp(a). Second, no correlation should exist between instrumental variables (IVs) and confounders. Third, the relationship between IVs and outcomes can only be through the exposure factor Lp(a). We considered whether the third hypothesis would be influenced by horizontal pleiotropy (IVs directly affecting the outcome) or by other recognized etiologies affecting CVD, excluding these limiting hypotheses (Fig. 1).

## Study population

### Lp(a) GWAS datasets

Single nucleotide polymorphisms (SNPs) were identified as IVs in our study. To obtain exposure group data, we chose SNPs closely associated with Lp(a) levels at the genome-wide significance level (*P* < 5E−8) from the GWAS including 377,590 European ancestors from Neale Lab. To avoid bias caused by linkage disequilibrium relationships in the analysis, the linkage disequilibrium of SNP, must satisfy the conditions *r*^*2*^ < 0.001 and *Kb* = 10,000. SNPs associated with CHD, MI, body mass index (BMI), and smoking were identified as multipotent IVs for AA, AF, CHD, and HF (genetic variation associated with multiple variables) [21–25], while SNPs associated with BMI were identified as IVs with pleiotropy for IS, LIS, ISL, ISS, PE, and HBP [26–28]. Extraction of information on IVs related to the exposure factor Lp(a) (Table 1). We estimated the *R*^2^ of each instrument and calculated the F statistic overall [29, 30]. The percentage of IVs that referred to exposure factors was what *R*^2^ refers to. When *F* value < 10, a weak instrumental variable was defined by reference to the value of *F* [31]:

$$R^{2} = 2 \times \left( {1 - {\text{MAF}}} \right) \times {\text{MAF}} \times \left( {\frac{\beta }{{{\text{SD}}}}} \right)^{2} ,$$

$$F = \left( {N - K - 1} \right)/K \times \frac{{R^{2} \,}}{{1 - R^{2} \,}}.$$

Description: MAF: minor allele frequency; SD = se.exposure $\times \surd N$; $\beta :$ *beta.* Exposure; *N*:no. of samples; *K*: no. of SNP.

**Table 1:** Extraction of information on instrumental variables related to the exposure factor Lp(a)

SNP	Effect_allele	Other_allele	Beta	eaf	se	*P*	*F*
rs71565789	C	T	−5.0482	0.021	0.473	1.364E−26	4.772
rs117733303	G	A	43.396	0.008	0.732	1E−200	57.929
rs146534110	T	G	28.147	0.013	0.597	1E−200	55.302
rs1086567	A	G	2.8444	0.626	0.138	8.983E−94	197.929
rs185864730	C	T	−10.317	0.014	0.582	3.276E−70	8.885
rs142709465	T	C	−4.2508	0.021	0.471	1.683E−19	3.332
rs3127580	T	C	27.895	0.157	0.177	1E−200	6713.695
rs12179053	T	C	−9.2946	0.259	0.152	1E−200	1448.551
rs184158723	A	G	18.557	0.019	0.495	1E−200	51.219
rs117857195	T	G	−16.547	0.023	0.477	1E−200	54.796
rs112110249	C	T	−10.644	0.043	0.329	1E−200	87.014
rs117881880	A	T	−13.415	0.015	0.557	5.64E−128	17.147
rs494554	G	C	−9.6137	0.027	0.426	9.89E−113	27.003
rs149210101	A	C	−10.149	0.019	0.497	1.911E−92	15.897
rs9355328	C	T	6.1439	0.975	0.432	5.955E−46	10.051
rs138581538	T	C	−8.2755	0.008	0.803	6.676E−25	1.727
rs10455872	G	A	89.361	0.072	0.204	1E−200	27,447.45
rs73596816	A	G	32.349	0.035	0.359	1E−200	552.619
Total						0.092089	118.093

### Different CVD GWAS datasets

Complete summary of statistical results in genome-wide association studies for AA (*n* = 209,366), CHD (n = 361,194), ISL (*n* = 150,765), AF (*n* = 1,030,836), HBP (*n* = 164,147), HF (*n* = 208,178), IS (*n* = 218,792), and LIS (*n* = 225,419), ISS (*n* = 198,048), PE (*n* = 218,413), and the above outcomes regarding CVD were based on European ancestry. Table 2 and Additional file 5: Table S5 show the SNPs for lipoprotein(a) and the SNPs and sources for each CVD, respectively, as well as the MR framework.

**Table 2:** The SNPs for lipoprotein(a) and the SNPs and sources for each cardiovascular disease

	No. of samples	No. of SNPs	Consortium/ID	Ancestry
Lp(a)	377,590	18	Neale Lab	European
AA	209,366	11	finn-b-I9_STR_EXH	European
CHD	361,194	11	finn-b-I9_CHD	European
ISL	150,765	18	ebi-a-GCST005840	European
AF	1,030,836	14	ebi-a-GCST006414	European
HBP	164,147	16	finn-b-I9_HYPTENSEC	European
HF	208,178	12	finn-b-I9_HEARTFAIL	European
IS	218,792	16	finn-b-I9_STR_EXH	European
LIS	225,419	13	ebi-a-GCST90014122	European
ISS	198,048	18	ebi-a-GCST006909	European
PE	218,413	16	finn-b-I9_PULMEMB	European

*AA* aortic aneurysm, *CHD* coronary heart disease, *ISL* large artery atherosclerosis stroke *AF* atrial fibrillation, *HBP* secondary hypertension, *HF* heart failure, *IS* ischemic stroke, *LIS* lacunar stroke, *ISS* small vessel stroke, *PE* pulmonary embolism

### Statistical analysis

To determine the causal relationships between Lp(a) levels and various CVD, MR-Egger, weighted median, random-effects inverse variance weighting (IVW1), maximum likelihood, penalized weighted median, and fixed-effects model inverse variance weighting (IVW2) were performed. For different validity assumptions, different Mendelian estimates can be derived from the aforementioned methods, the most prominent of which is the IVW1 because all of its SNPs are valid IVs and the method produces accurate estimates [32], In the primary analyses, odds ratio (OR) estimates with 95% confidence intervals (CI) were reported. We would have more confidence in inferring causality and opposing multiplicity (or other forms of bias) if all of the above methods were consistent. Mendelian randomization-PRESSO (MR-PRESSO) offers an alternative statistical detection method. Due to the bias of the multieffectiveness (global test) and the provision of an accurate estimate by outlier kick-out [33]. Briefly, MR-PRESSO corrects for horizontal pleiotropy by removing outliers. To assess heterogeneity, we utilized MR-Egger regression and IVW. The MR-Egger interaction method was used to test for horizontal pleiotropy and the leave-one-out method was used to investigate the possibility that this causal relationship was driven by a single SNP. Anderson–Darling normality test and Shapiro–Wilk normality test were used to test for normality. All statistical tests were two-tailed, and a *P* < 0.05 was considered statistically significant. All data analysis was implemented using R Studio 4.2.1 with the “ Two-Sample-MR (version 0.5.6, Bristol, UK)” “ MR-PRESSO (version 1.0, New York, NY, USA)” and “ Mr. raps” packages for MR analysis.

## Results

### Selection of instrumental variable

We chose diverse numbers of SNPs as IVs for various CVD. 11, 14, 11, 18, 16, 12, 16,13, 18, and 16 was selected as IVs for AA, AF, CHD, ISL, HBP, HF, IS, LIS, ISS, and PE, respectively. From the scope of the GWAS, they were all associated with Lp(a) levels. When the value of F > 10, a strong IV is defined with reference to the value of F (Table 1).

### The causal effect between Lp(a) and different CVD

Patients with high Lp(a) levels has a 0.5-fold increased risk of AA(OR = 1.005, 95% CI 1.001–1.010, *P* = 0.009), a 0.3-fold increased risk of CHD (OR = 1.003, 95% CI 1.001–1.004, *P* = 0.010) and a 0.2-fold increased risk of ISL (OR = 1.003, 95% CI 1.002–1.004, *P* = 9.50E−11) using the IVW1. The IVW1 estimate showed that AF (OR = 1.001, 95% CI 1.000–1.002, *P* = 0.097), IS(OR = 1.001, 95% CI 1.000–1.001, *P* = 0.156), LIS(OR = 1.000, 95% CI 1.000–1.001, *P* = 0.524), PE(OR = 1.000, 95% CI 0.998–1.000, *P* = 0.210), HBP(OR = 1.000, 95% CI 0.998 1.002, *P* = 0.927), ISS (OR = 0.999, 95% CI 0.998–1.001, *P* = 0.430), HF(OR = 0.999, 95% CI 0.997–1.002, *P* = 0.584) were not associated with Lp(a). The MR-Egger estimate showed that genetically predicted Lp(a) was not significantly associated with the risk of CHD (Table 3). For AA, MR-PRESSO yielded *P* = 0.081, but no outliers were identified; therefore, The IVW1 method is more reliable. Table 4 displays the MR-PRESSO conclusions. There were no directional pleiotropies for the analysis results (all *P* > 0.05) (Table 3). Anderson–Darling normality test and Shapiro–Wilk normality test showed that only HF at the Anderson–Darling normality test was not normally distributed (*P* = 0.041), but the Shapiro–Wilk normality test was predominant.

**Table 3:** Association between plasma lipoprotein a levels and cardiovascular diseases in Mendelian randomization analysis (six different methods corresponding to the results), heterogeneity as well as horizontal multiplicity test analysis

Outcome	MR methods	OR (95% CI)	*P* for association	*P* for heterogeneity test	*P* for MR-Egger intercept
AA	MR-Egger	1.010 (1.002–1.019)	0.038	0.623	0.220
	IVW1	1.005 (1.001–1.010)	0.009	0.544	
	Weighted median	1.006 (1.000–1.011)	0.039		
	Maximum likelihood	1.006 (1.001–1.010)	0.009		
	PWM	1.006 (1.000–1.011)	0.042		
	IVW2	1.005 (1.001–1.010)	0.009		
CHD	MR-Egger	0.999 (0.992–1.005)	0.723	0.753	0.271
	IVW1	1.003 (1.001–1.004)	0.010	0.703	
	Weighted median	1.003 (1.001–1.006)	0.014		
	Maximum likelihood	1.003 (1.001–1.004)	0.010		
	PWM	1.003 (1.001–1.006)	0.011		
	IVW2	1.003 (1.001–1.004)	0.010		
ISL	MR-Egger	1.003 (1.001–1.004)	3.56E-04	0.588	0.381
	IVW1	1.003 (1.002–1.004)	9.50E-11	0.599	
	Weighted median	1.003 (1.002–1.004)	1.05E-07		
	Maximum likelihood	1.003 (1.002–1.004)	9.10E-11		
	PWM	1.003 (1.002–1.004)	2.28E-07		
	IVW2	1.003 (1.002–1.004)	9.50E-11		
AF	MR-Egger	1.002 (1.000–1.005)	0.070	0.829	0.204
	IVW1	1.001 (1.000–1.002)	0.097	0.756	
	Weighted median	1.001 (1.000–1.003)	0.089		
	Maximum likelihood	1.001 (1.000–1.002)	0.098		
	PWM	1.001 (1.000–1.002)	0.083		
	IVW2	1.001 (1.000–1.002)	0.097		
HBP	MR-Egger	1.000 (0.998–1.002)	0.997	0.457	0.900
	IVW1	1.000 (0.998–1.002)	0.927	0.532	
	Weighted median	1.000 (0.998–1.002)	0.892		
	Maximum likelihood	1.000 (0.998–1.002)	0.927		
	PWM	1.000 (0.998–1.002)	0.894		
	IVW2	1.000 (0.998–1.002)	0.927		
HF	MR-Egger	1.000 (0.994–1.005)	0.883	0.060	0.909
	IVW1	0.999 (0.997–1.002)	0.584	0.088	
	Weighted median	1.000 (0.997–1.003)	0.917		
	Maximum likelihood	0.999 (0.997–1.001)	0.489		
	PWM	1.000 (0.997–1.003)	0.921		
	IVW2	0.999 (0.997–1.001)	0.488		
IS	MR-Egger	1.001 (1.000–1.002)	0.152	0.197	0.514
	IVW1	1.001 (1.000–1.001)	0.156	0.223	
	Weighted median	1.001 (1.000–1.001)	0.069		
	Maximum likelihood	1.001 (1.000–1.001)	0.112		
	PWM	1.001 (1.000–1.001)	0.698		
	IVW2	1.001 (1.000–1.001)	0.112		
LIS	MR-Egger	1.000 (1.000–1.001)	0.847	0.159	0.736
	IVW1	1.000 (1.000–1.001)	0.524	0.205	
	Weighted median	1.000 (0.999–1.000)	0.331		
	Maximum likelihood	1.000 (0.999–1.000)	0.466		
	PWM	1.000 (0.999–1.000)	0.314		
	IVW2	1.000 (0.999–1.000)	0.466		
ISS	MR-Egger	0.999 (0.998–1.001)	0.353	0.007	0.381
	IVW1	0.999 (0.998–1.001)	0.430	0.009	
	Weighted median	0.999 (0.998–1.000)	0.071		
	Maximum likelihood	0.999 (0.999–1.000)	0.265		
	PWM	0.999 (0.998–1.000)	0.723		
	IVW2	0.999 (0.999–1.000)	0.265		
PE	MR-Egger	1.000 (0.998–1.001)	0.826	0.240	0.204
	IVW	1.000 (0.998–1.000)	0.210	0.192	
	Weighted median	1.000 (0.999–1.001)	0.835		
	Maximum likelihood	0.999 (0.998–1.000)	0.152		
	PWM	1.000 (0.999–1.001)	0.879		
	IVW2	0.999 (0.998–1.000)	0.152		

*IVW1* random-effects inverse variance weighting, *IVW2* fixed-effects model inverse variance weighting, *PWM* penalized weighted median, *OR* odds ratio, *CI* confidence interval, *AA* aortic aneurysm *CHD* coronary heart disease. *ISL* large artery atherosclerosis stroke, *AF* atrial fibrillation, *HBP* secondary hypertension, *HF* heart failure, *IS* ischemic stroke, *LIS* lacunar stroke, *ISS* small vessel stroke, *PE* pulmonary embolism

**Table 4:** Association of plasma Lp(a) levels with cardiovascular disease in a Mendelian randomization analysis (MR-PRESSO)

	*N*	RAW OR	95% CI	Estimates *P*	outlier *N*	OR	Corrected 95% CI	Estimates *P*
AA	11	1.005	1.000–1.009	0.081	NA	NA	NA	NA
CHD	11	1.002	1.001–1.005	0.028	NA	NA	NA	NA
ISL	18	1.003	1.002–1.004	1.40E-06	NA	NA	NA	NA
AF	14	1.001	1.000–1.002	0.081	NA	NA	NA	NA
HBP	16	1.000	0.998–1.002	0.915	NA	NA	NA	NA
HF	12	0.999	0.997–1.002	0.564	NA	NA	NA	NA
IS	16	1.000	1.000–1.001	0.162	NA	NA	NA	NA
LIS	13	1.000	0.999–1.000	0.547	NA	NA	NA	NA
ISS	18	0.999	0.998–1.000	0.445	NA	NA	NA	NA
PE	16	0.999	0.998–1.000	0.228	NA	NA	NA	NA

*OR* odds ratio, *CI* confidence interval, *AA* aortic aneurysm, *CHD* coronary heart disease, *ISL* large artery atherosclerosis stroke, *AF* atrial fibrillation, *HBP* secondary hypertension *HF* heart failure, *IS* ischemic stroke, *LIS* lacunar stroke. *ISS* small vessel stroke, *PE* pulmonary embolism

### Heterogeneity and sensitivity

**Fig. 2:**
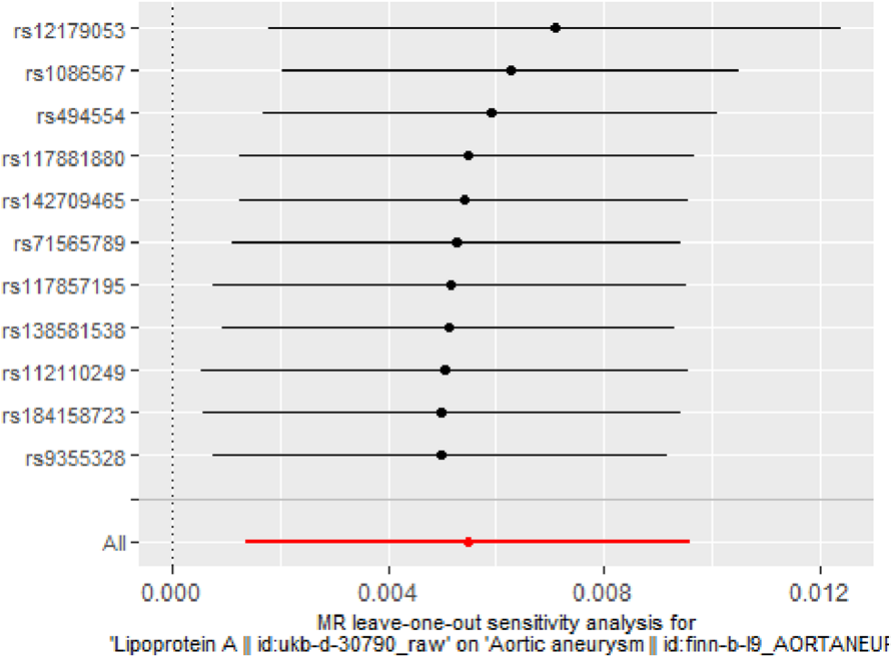
MR leave-one-out sensitivity analysis for Lp(a) on AA

**Fig. 3:**
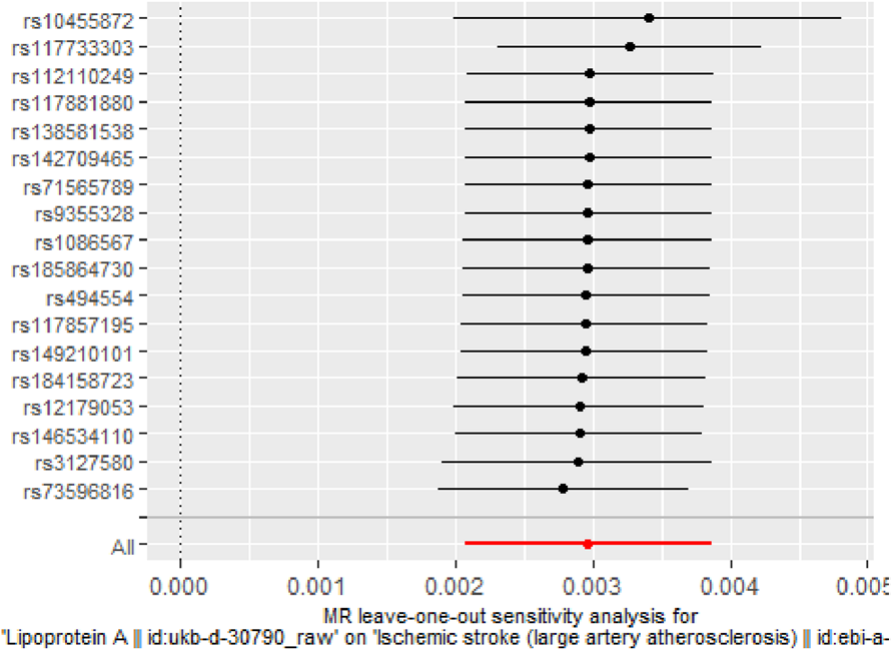
MR leave-one-out sensitivity analysis for Lp(a) on ISL

MR-Egger regression revealed heterogeneity for ISS (*P* = 0.007), whereas the IVW revealed heterogeneity for ISS (*P* = 0.009). The scatter plots and forest plots are displayed in Additional file 1: Figure S1 and Additional file 2: Figure S2. The funnel plots were symmetrical (Additional file 3: Figure S3) and the leave-one-out method indicated that no SNP was substantially driving the association between lipids traits and CVD risks (Figs. 2, 3 and Additional file 4: Figure S4).

From the above analysis, it is evident that there is a causal relationship between Lp(a) and AA, CHD, and ISL, but not with other CVDs.

## Discussion

This is one of the largest MR analysis to study the effect of Lp(a) on different CVDs such as AA, CHD, ISL, AF, HBP, HF, IS, LIS, ISS, and PE. Our study provided additional evidence that lowering Lp(a) will reduce the prevalence of CVD and may contribute to a better understanding of the genetic impact of Lp(a) on CVD. Our study suggested that patients with high Lp(a) levels has a 0.5-fold increased risk of AA(OR = 1.005, 95% CI 1.001–1.010, *P* = 0.009), with potential mechanisms including atherosclerosis and promotion of inflammation [34, 35], which all increased the risk of AA. It was demonstrated that elevated Lp(a) concentrations were independently associated with an increased risk of abdominal aortic aneurysms (AAA) in a population-based cohort study [36], and a meta-analysis demonstrated that high levels of Lp(a) may be linked to the presence of AAA and that Lp(a) may be a marker to screen for AAA [37]. Our study may provide more reliable evidence for a causal relationship between Lp(a) and CHD, and the relationship with CHD is similar to the findings previously observed in CHD Exome + (odds ratio rescaled per 50 mg/dL increment of Lp(a) levels, 1.35 [95% CI 1.29–1.41]) [38]. According to a research, Lp(a) is related with rapid advancement of coronary plaques, which may explain the elevated residual risk of MI associated with Lp(a) [39]. Lp(a) was independently associated with ISL [12], and we found a 0.2-fold increased risk of ISL(OR = 1.003, 95% CI 1.002–1.004, *P* = 9.50E−11). It has been shown that elevated serum Lp(a) levels predict the risk of early stroke recurrence in patients with a first IS [40], but we concluded that there is null association between Lp(a) and IS, LIS and ISS. The unique structure of the Lp(a) may form the link between atherosclerosis (which is partially mediated by the LDL sample) and thrombotic (partially mediated by apolipoprotein B), and contribute to IS [41]. In addition, heterogeneity was observed in the analysis of Lp(a) and ISS, which may be related to age and gender, but we were unable to obtain relevant genome-wide association subgroup information at this time. There was no correlation between Lp(a) and AF, HF, or PE. However, the findings of a previous study suggested that adjusted Lp(a) was predictive of new-onset AF (hazard ratio for 1-SD increase, 2.69; 95% CI 1.00–7.22; *P* < 0.05) [42]. In contrast, a MR study suggested that Lp(a) may be a potential causative risk factor for AF, which requires confirmation in a large number of future investigations [43]. There have been few studies on Lp(a) and HBP, and it is clear that there is no connection between Lp(a) and HBP, evidence from a clinic indicates that hypertensive cohort's patients had increased Lp(a) levels, indicating that Lp(a) assessment may be helpful in risk stratification [14]. This suggested that more research will be required in this field. There was no link between Lp(a) and HF, and a community research found no correlation between Lp(a) and HF, too; yet, some studies implied that Lp(a) increased the risk of HF [13]; therefore, larger investigations are required. Lp(a) had a structural component comparable to fibrinogen, and its oxidative, pro-inflammatory actions may be related with venous thrombosis leading to PE; nevertheless, our investigation found no association between Lp(a) and the incidence of PE events. Similar investigations also failed to find a link between PE severity and Lp(a) levels [44].

The main advantages of this study were the application of a MR analysis that can resist confounding factors and the use of a large GWAS to reduce the possibility of false negatives. The results of MR investigations, however, were susceptible to pleiotropy [45]. By removing specific genetic variants from the current investigation, we were able to lower the pleiotropy of genetic variants. There were certain restrictions, though. First, MR relies on three main assumptions that were difficult to verify empirically. In addition, we did not investigate the causal relationship between other lipids and CVD because we focused primarily on genetically determined Lp(a), whose causative mechanisms remain controversial and whose effects on a variety of CVD were also debatable. To further justify the use of lipid-lowering drugs, the following study might use a multivariate MR technique to examine the impact of various lipids or lipoproteins on CVD. A MR analysis based on different population groups should be conducted to eliminate racial bias, as Lp(a) concentrations are mostly genetically determined and population-related. Furthermore, because we lacked complete information on our participants' clinical features and numerous sizable GWAS, we were unable to do subgroup analysis. Finally, given that future GWAS will always overlook people who have passed away from exposure or other competing risks for outcomes, our results may be biased due to selection.

## Conclusion

This MR study suggested a causal relationship between Lp(a) and CHD, AA, and ISL, but not associated with AF, HF, IS, LIS, ISS, HBP, or PE. Our work further verifies the association between Lp(a) and various CVD, resulting in improved Lp(a) management and a reduction in the prevalence of CVD.

## Supplementary Information

**Additional file 1: Figure S1.** The scatter plots of Lp(a) on CVD.

**Additional file 2: Figure S2.** The forest plots of Lp(a) on CVD.

**Additional file 3: Figure S3.** The funnel plots of Lp(a) on CVD.

**Additional file 4: Figure S4.** The leave-one-out method of Lp(a) on CVD (except AA and ISL).

**Additional file 5:** The SNPs for each CVD.

## Data Availability

Not applicable.

## Abbreviations

Lp(a): Lipoprotein(a)
CVD: Cardiovascular disease
LDL-C: Low-density lipoprotein cholesterol
MR: Mendelian randomization
GWAS: Genome-wide association study
IVs: Instrumental variables
SNPs: Single nucleotide polymorphisms
AA: Aortic aneurysm
CHD: Coronary heart disease
ISL: Large artery atherosclerosis stroke
AF: Atrial fibrillation
HBP: Secondary hypertension
HF: Heart failure
IS: Ischemic stroke
LIS: Lacunar stroke
ISS: Small vessel stroke
PE: Pulmonary embolism
IVW1: Random-effects inverse variance weighting
IVW2: Fixed-effects model inverse variance weighting
PWM: Penalized weighted median
MR-PRESSO: Mendelian randomization-PRESSO
OR: Odds ratio
CI: Confidence interval
BMI: Body mass index
MI: Myocardial infarction
