# Supplementary material for: The relationship between lipoprotein(a) and risk of cardiovascular disease: a Mendelian randomization analysis
Source: Eur J Med Res. 2022 Oct 27;27:211. doi: 10.1186/s40001-022-00825-6 (PMC9608881; doi:10.1186/s40001-022-00825-6)
Supplement: Supplementary file 1 — Additional file 1: Figure S1. The scatter plots of Lp(a) on CVD. [file 40001_2022_825_MOESM1_ESM.pdf]

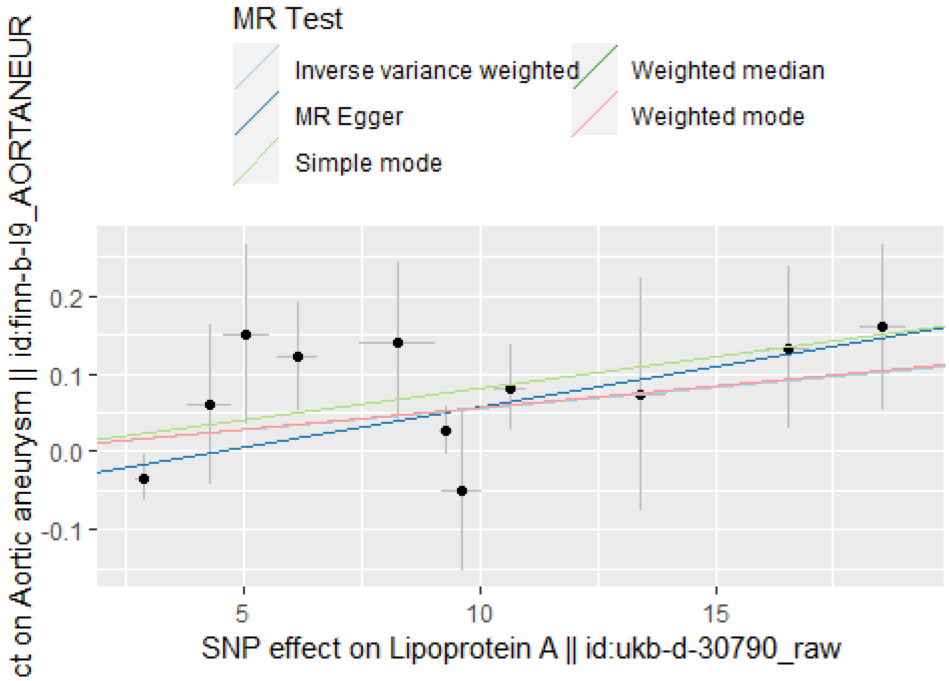
MR Test
Inverse variance weighted
MR Egger
Simple mode
Weighted median
Weighted mode
0.2
0.1
0.0
-0.1
5
10
15
SNP effect on Lipoprotein A || id:ukb-d-30790_raw
ct on Aortic aneurysm || id:finn-b-I9_AORTANEUR

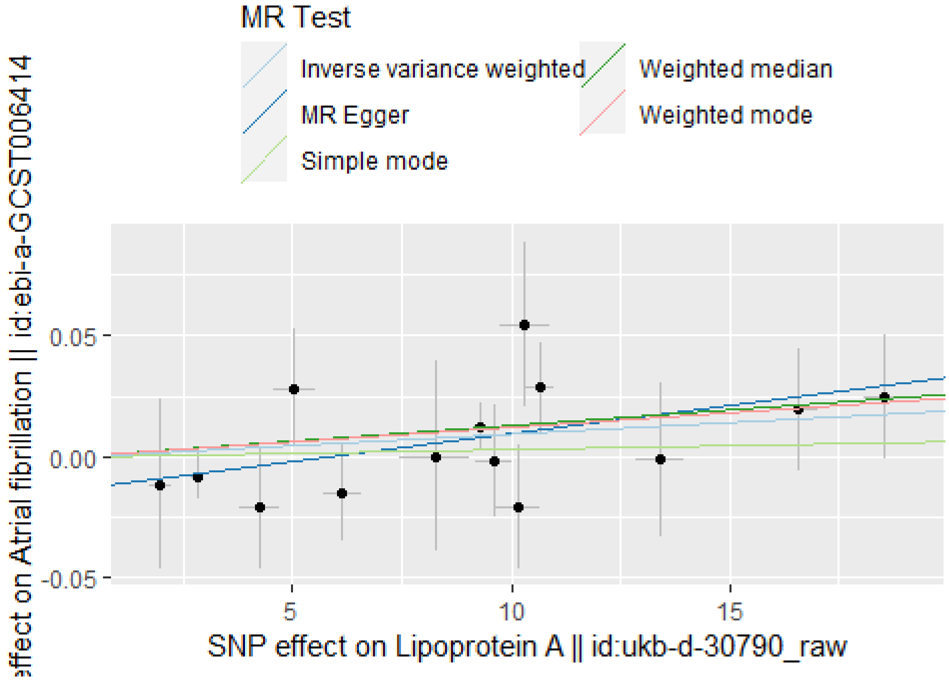
MR Test
Inverse variance weighted
MR Egger
Simple mode
Weighted median
Weighted mode
0.05
0.00
-0.05
5
10
15
SNP effect on Lipoprotein A || id:ukb-d-30790_raw
effect on Atrial fibrillation || id:ebi-a-GCST006414

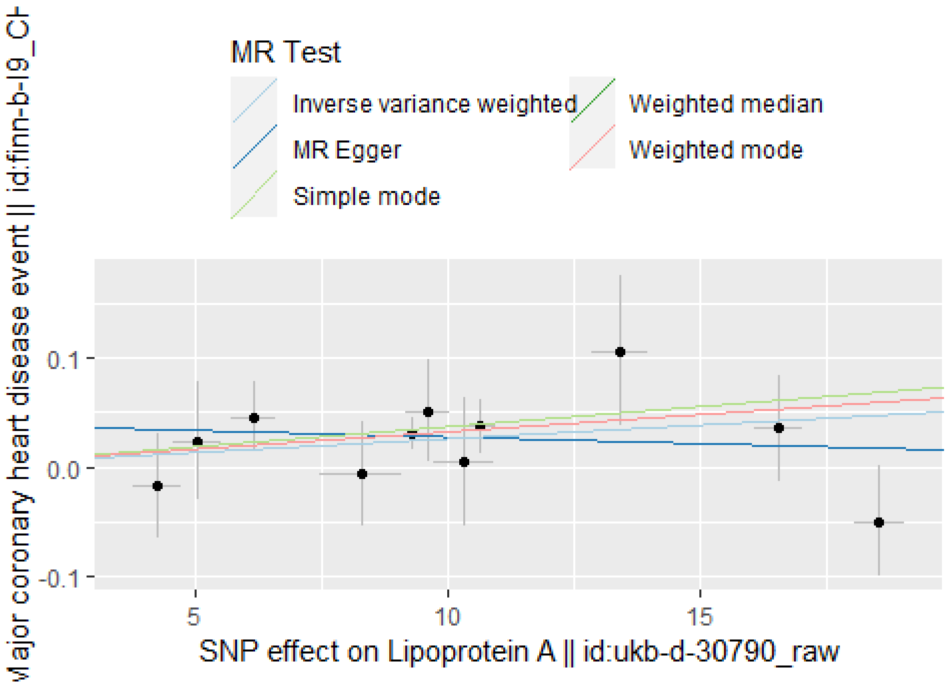

MR Test
Inverse variance weighted
MR Egger
Simple mode
Weighted median
Weighted mode
0.1
0.0
-0.1
5
10
15
SNP effect on Lipoprotein A || id:ukb-d-30790_raw
Major coronary heart disease event || id:finn-b-I9_CH

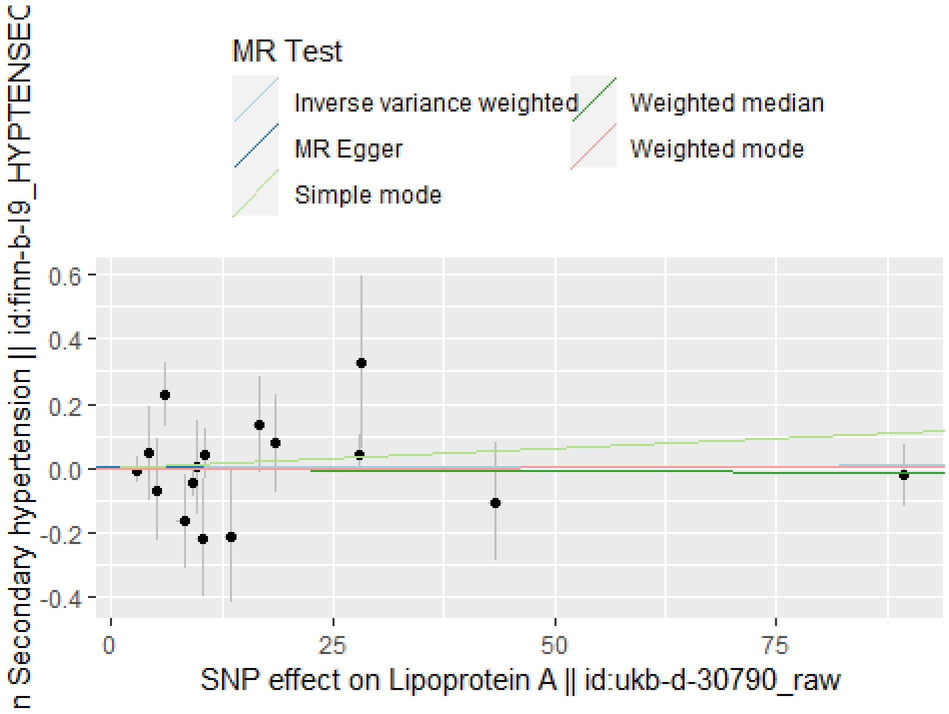

MR Test
Inverse variance weighted
MR Egger
Simple mode
Weighted median
Weighted mode
0.6
0.4
0.2
0.0
-0.2
-0.4
0
25
50
75
SNP effect on Lipoprotein A || id:ukb-d-30790_raw
n Secondary hypertension || id:finn-b-I9_HYPTENSEC

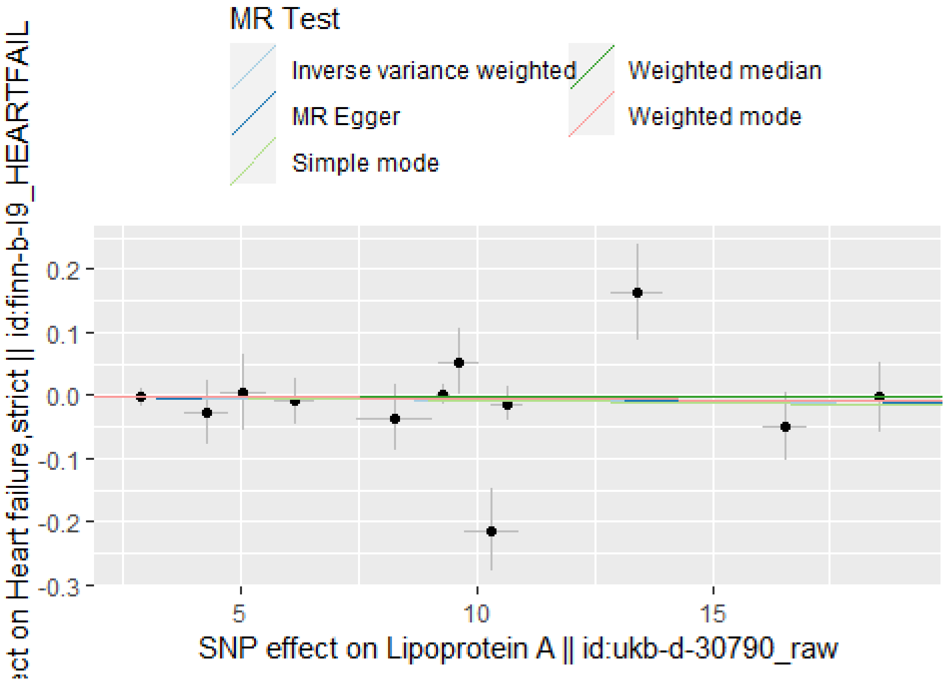
MR Test
Inverse variance weighted
MR Egger
Simple mode
Weighted median
Weighted mode
ect on Heart failure,strict || id:finn-b-I9_HEARTFAIL
0.2
0.1
0.0
-0.1
-0.2
-0.3
5
10
15
SNP effect on Lipoprotein A || id:ukb-d-30790_raw

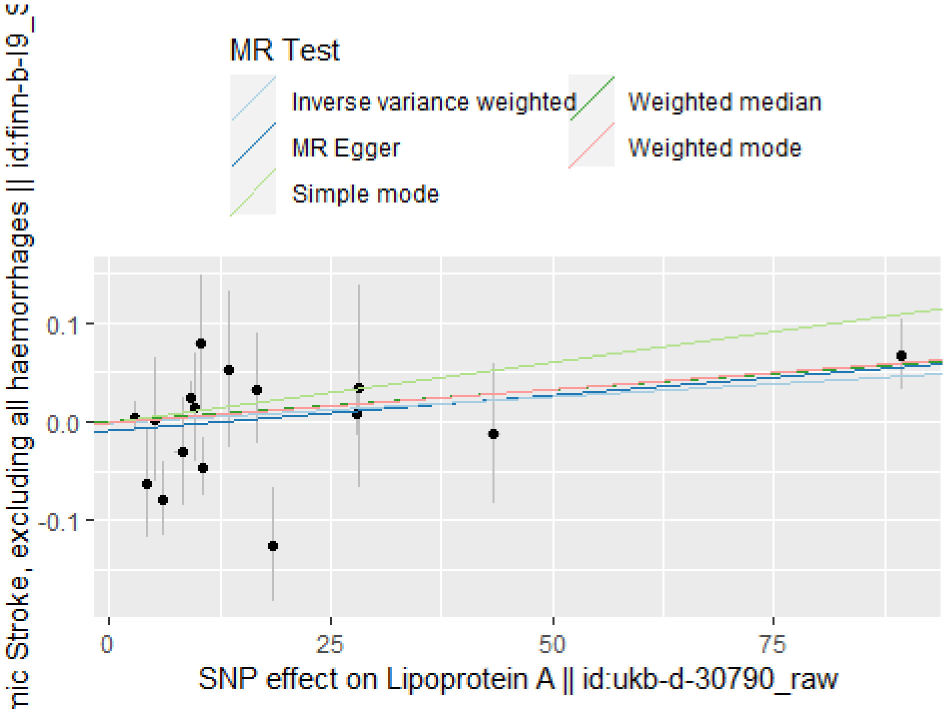
MR Test
Inverse variance weighted
MR Egger
Simple mode
Weighted median
Weighted mode
nic Stroke, excluding all haemorrhages || id:finn-b-I9_S
0.1
0.0
-0.1
0
25
50
75
SNP effect on Lipoprotein A || id:ukb-d-30790_raw

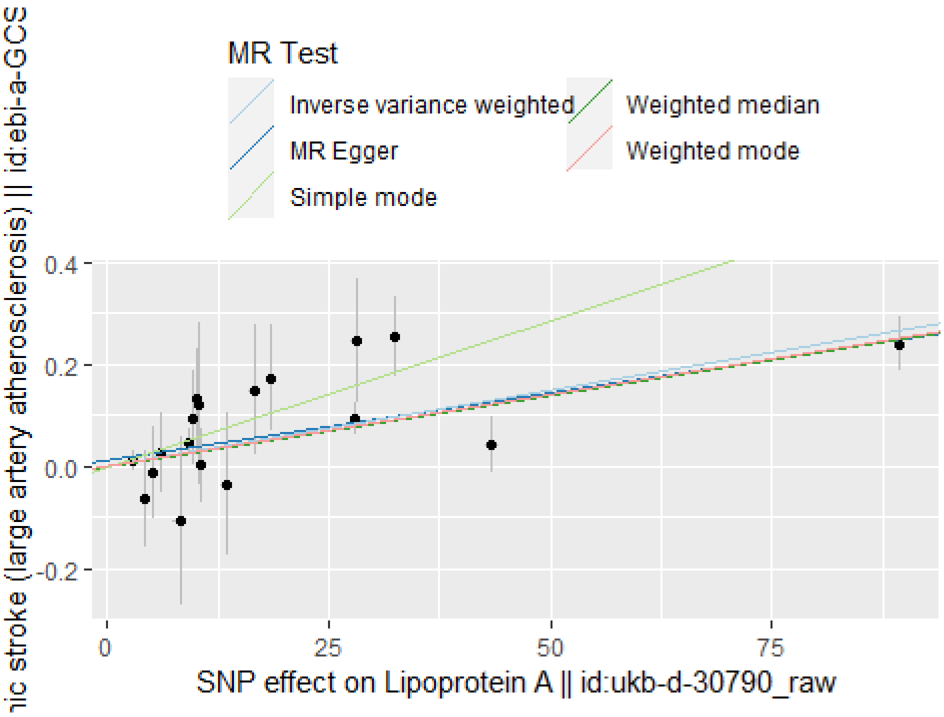
MR Test
Inverse variance weighted
MR Egger
Simple mode
Weighted median
Weighted mode
0.4
0.2
0.0
-0.2
0
25
50
75
SNP effect on Lipoprotein A || id:ukb-d-30790_raw
ic stroke (large artery atherosclerosis) || id:ebi-a-GCS

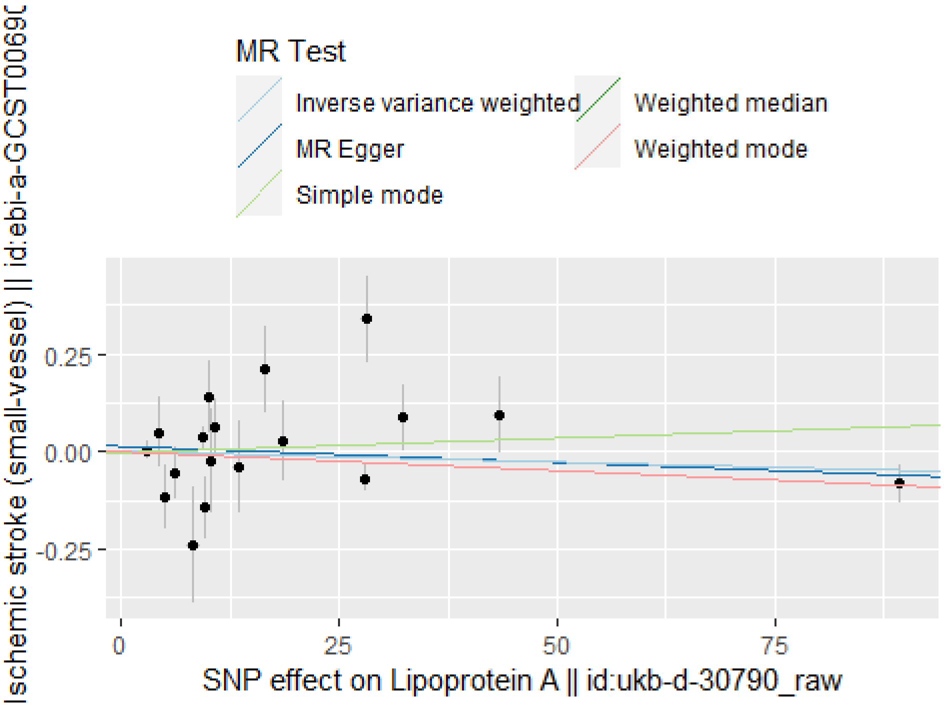
MR Test
Inverse variance weighted
MR Egger
Simple mode
Weighted median
Weighted mode
0.25
0.00
-0.25
0
25
50
75
SNP effect on Lipoprotein A || id:ukb-d-30790_raw
Ischemic stroke (small-vessel) || id:ebi-a-GCST0069

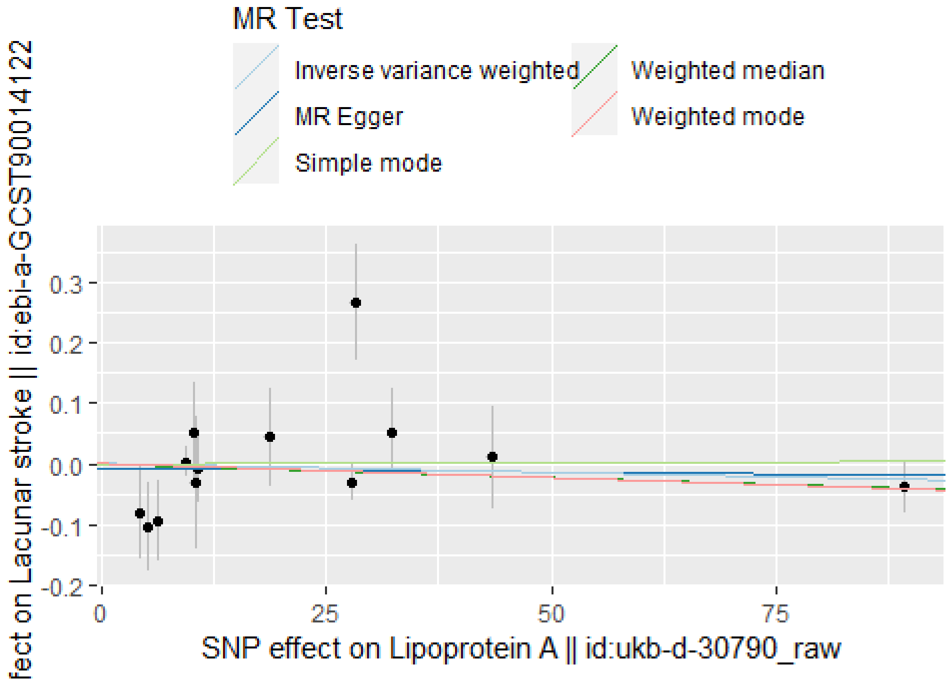
MR Test
Inverse variance weighted
MR Egger
Simple mode
Weighted median
Weighted mode
0.3
0.2
0.1
0.0
-0.1
-0.2
0
25
50
75
SNP effect on Lipoprotein A || id:ukb-d-30790_raw
fect on Lacunar stroke || id:ebi-a-GCST90014122

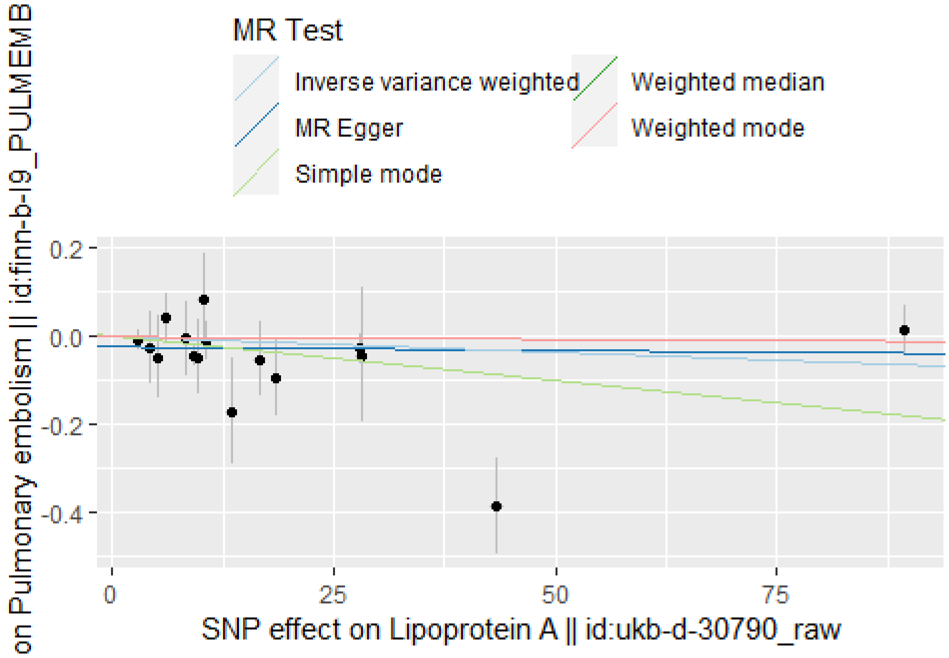
MR Test
Inverse variance weighted
MR Egger
Simple mode
Weighted median
Weighted mode
0.2
0.0
-0.2
-0.4
0
25
50
75
SNP effect on Lipoprotein A || id:ukb-d-30790_raw
on Pulmonary embolism || id:finn-b-I9_PULMEMB
