# Supplementary figures and images for: The relationship between lipoprotein(a) and risk of cardiovascular disease: a Mendelian randomization analysis
Source: Eur J Med Res. 2022 Oct 27;27:211. doi: 10.1186/s40001-022-00825-6 (PMC9608881; doi:10.1186/s40001-022-00825-6)

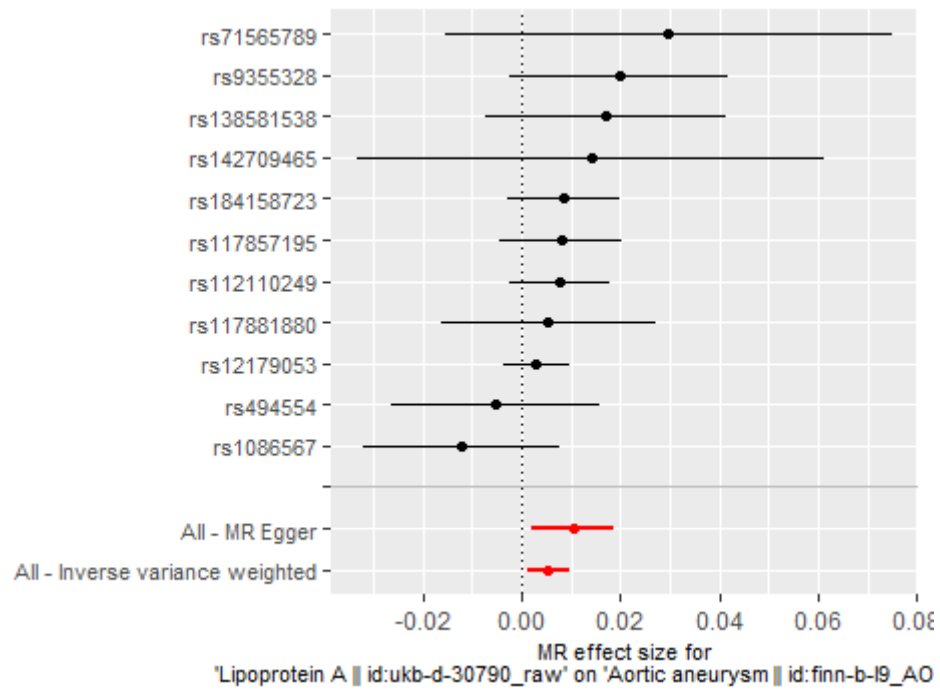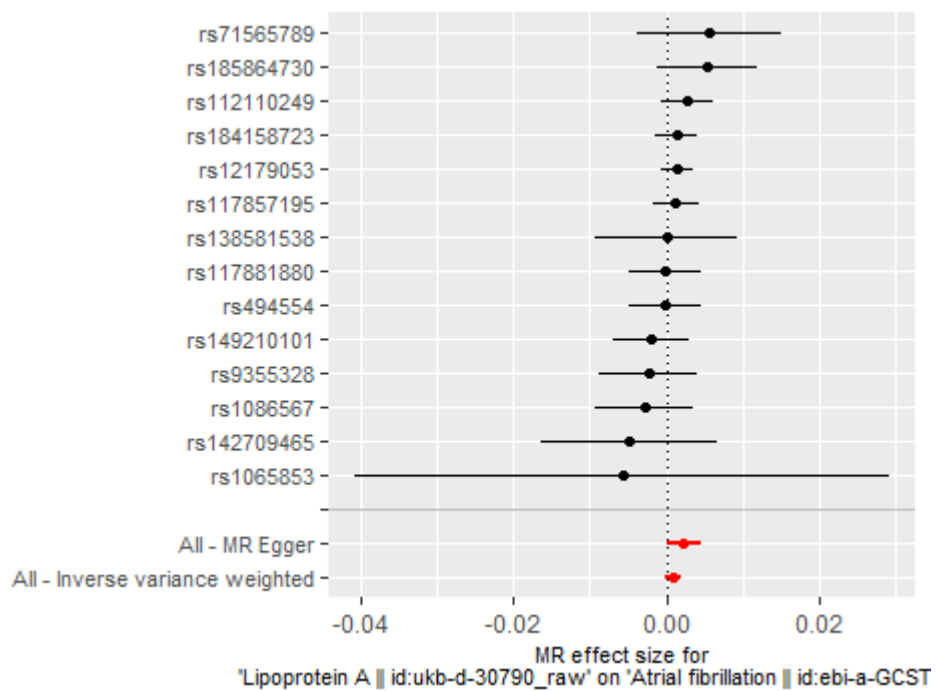

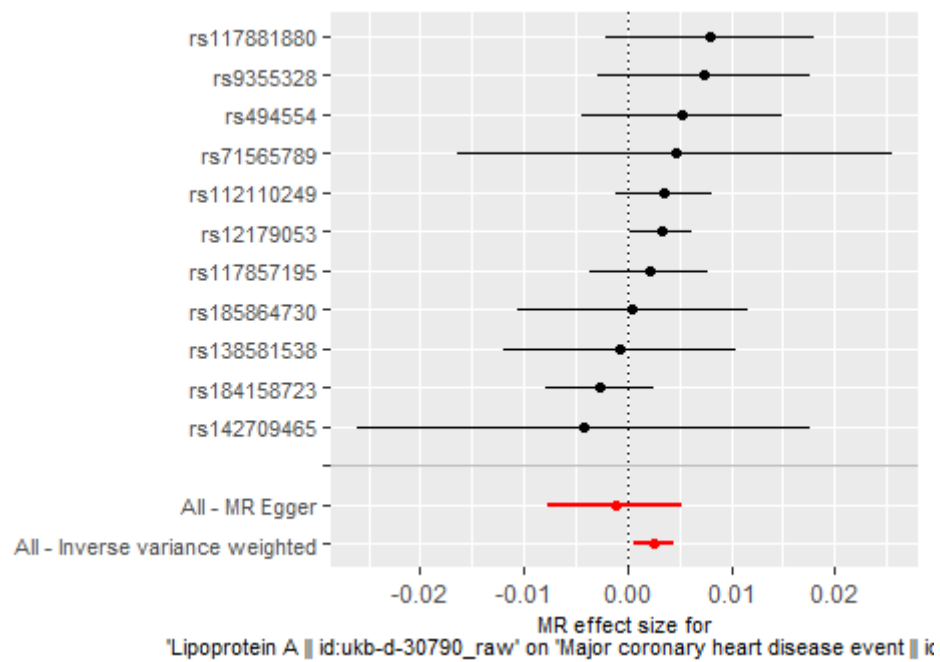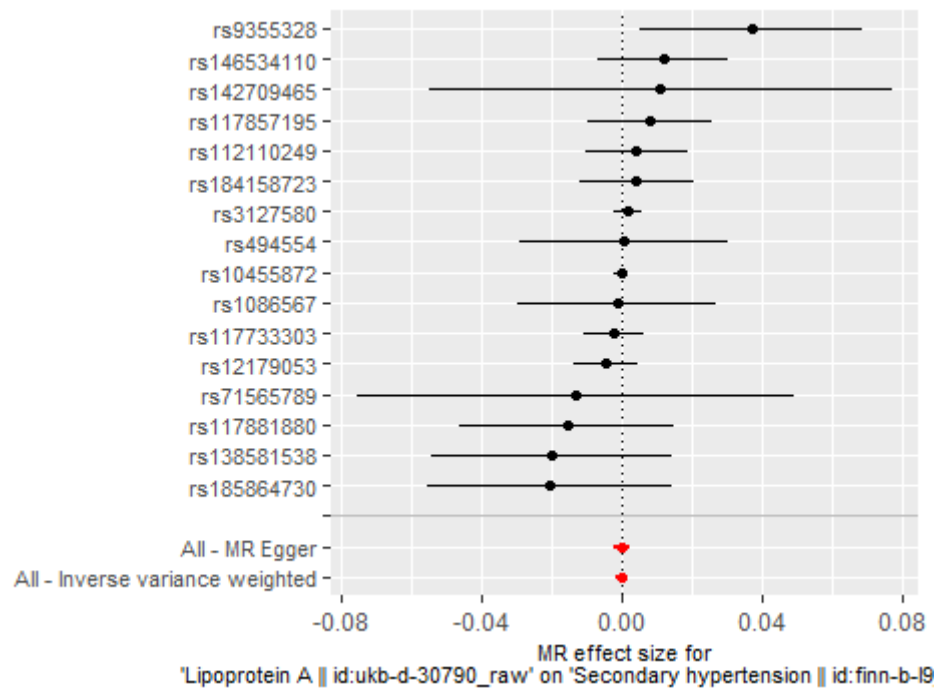

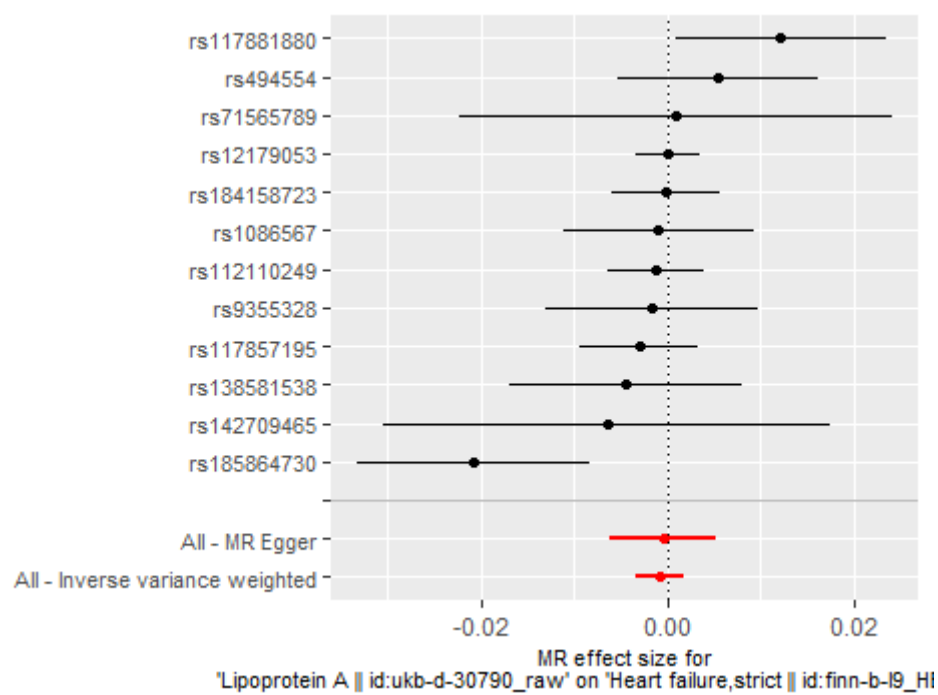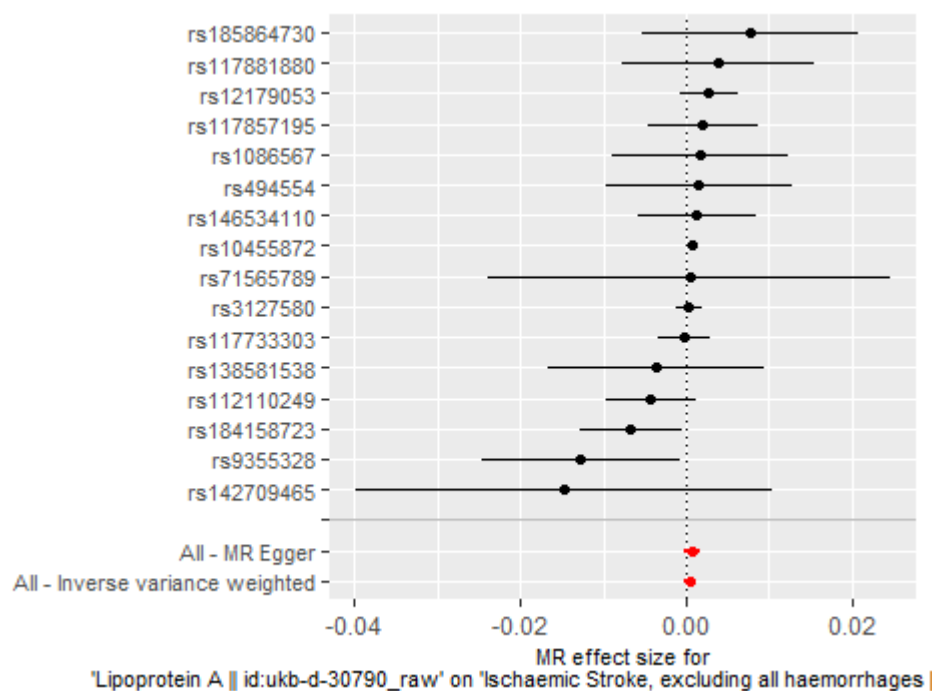

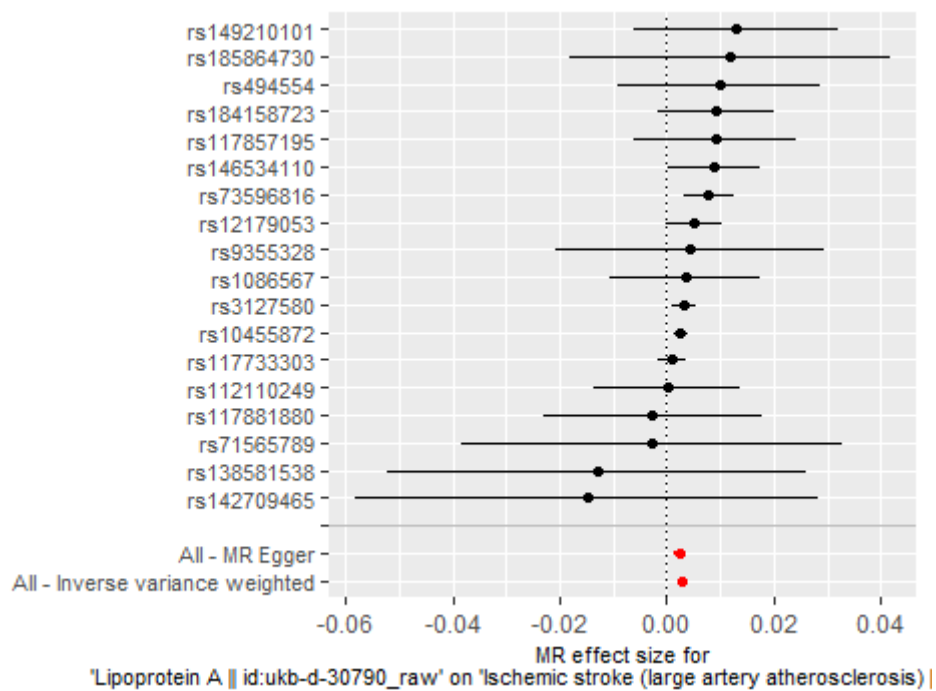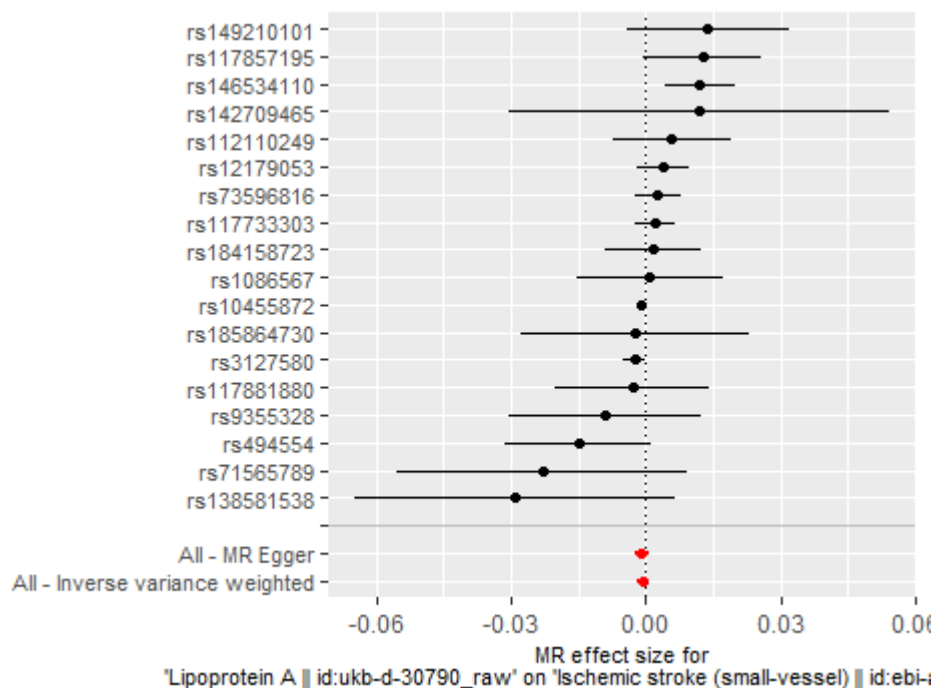

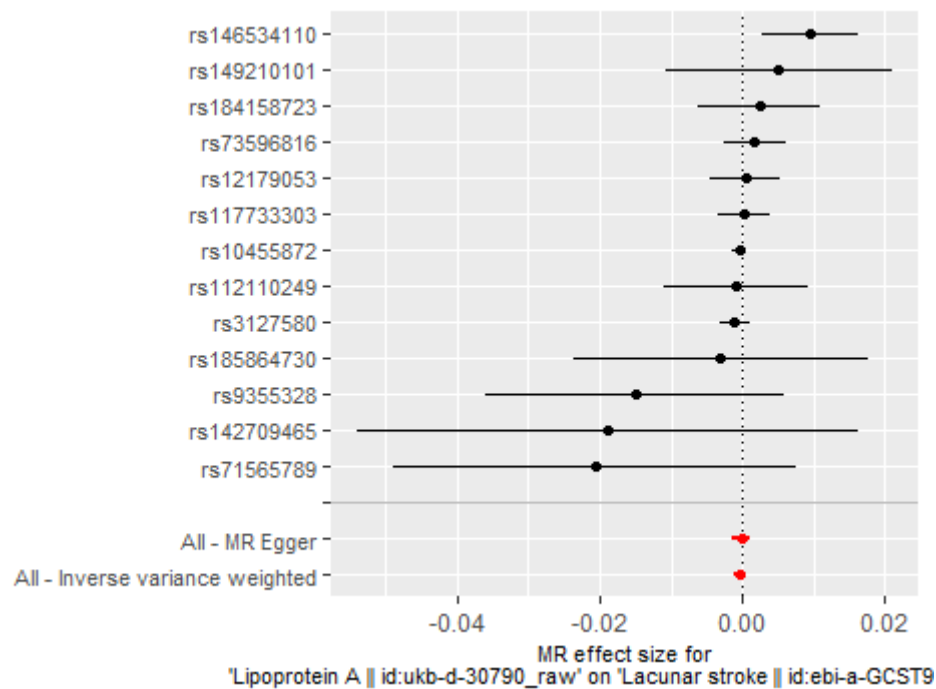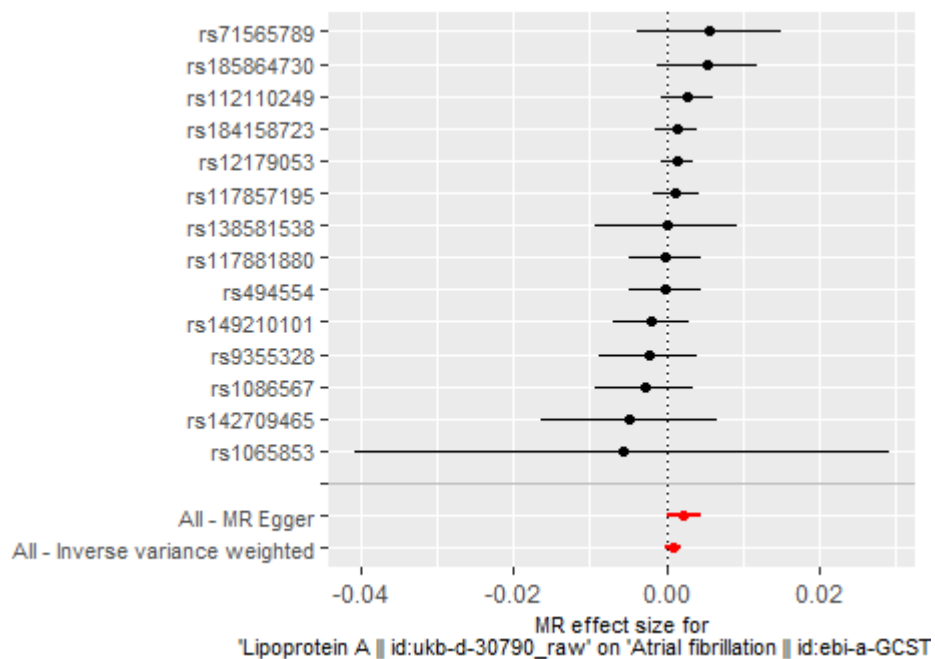

Supplement: Supplementary file 2 — Additional file 2: Figure S2. The forest plots of Lp(a) on CVD. [file 40001_2022_825_MOESM2_ESM.pdf]

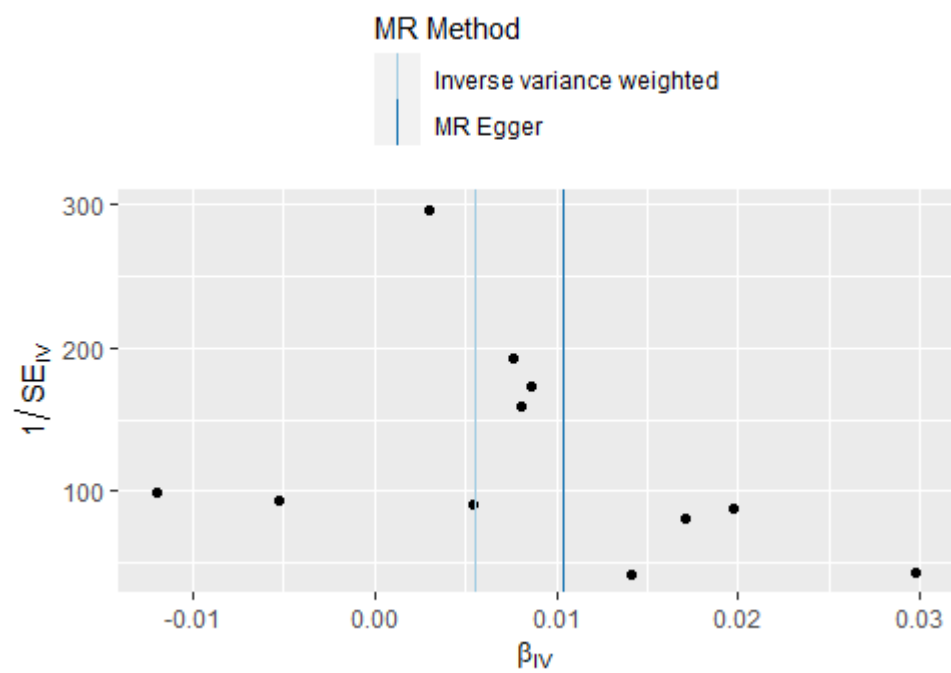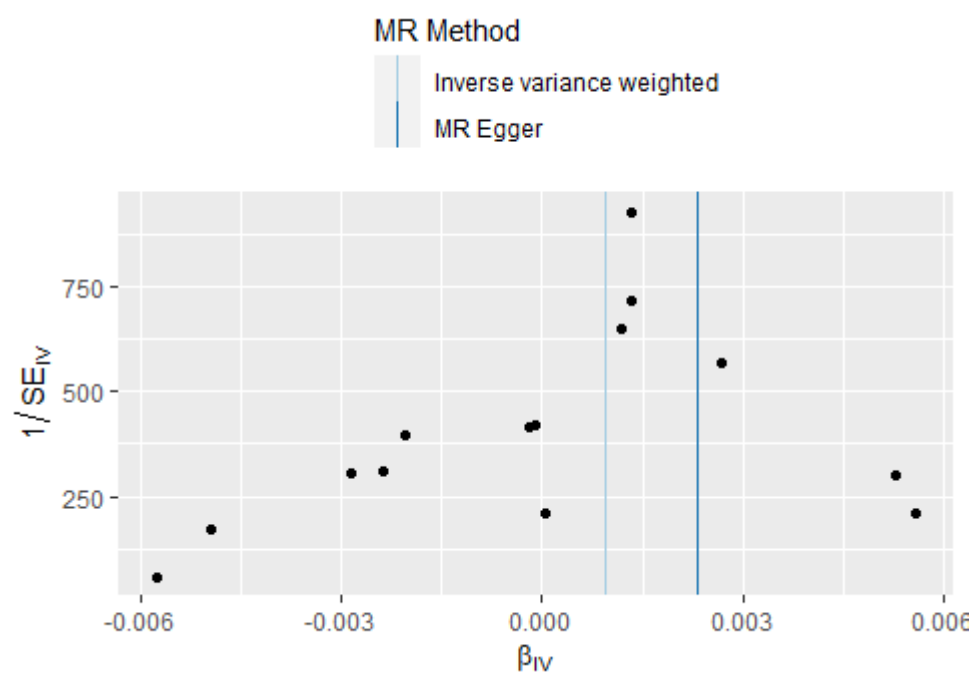

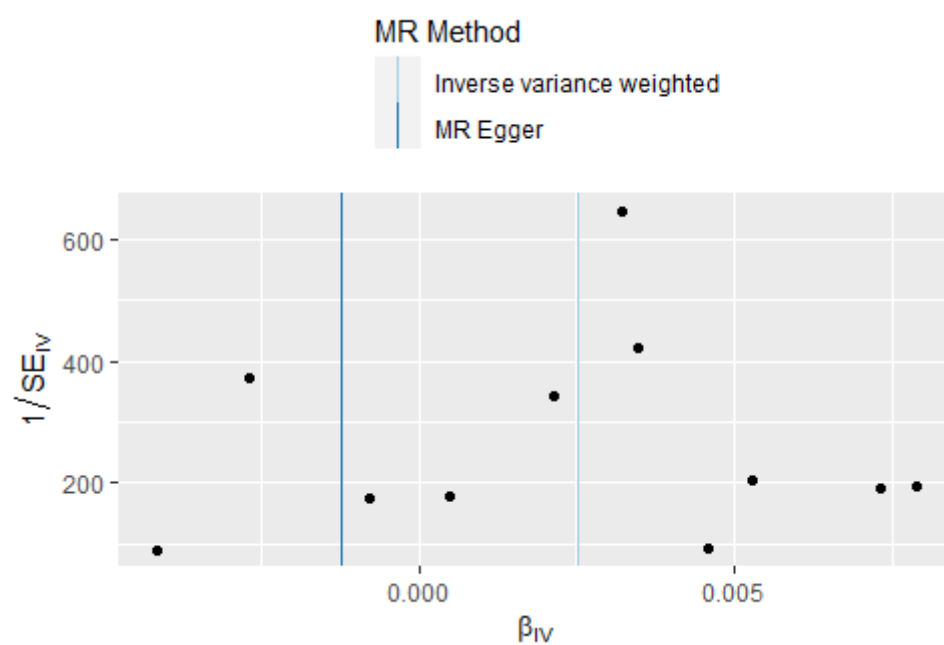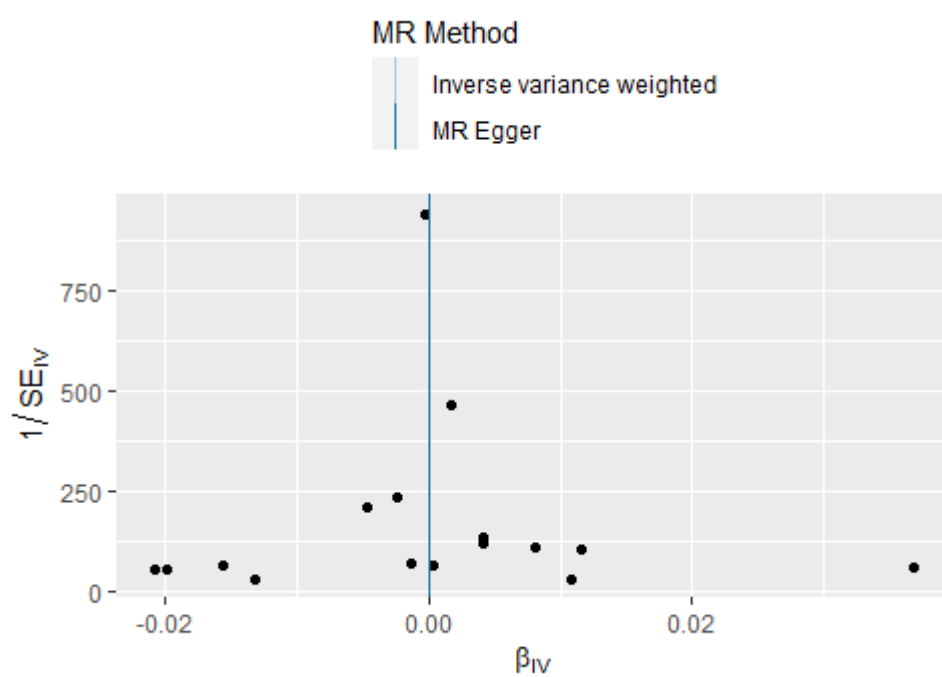

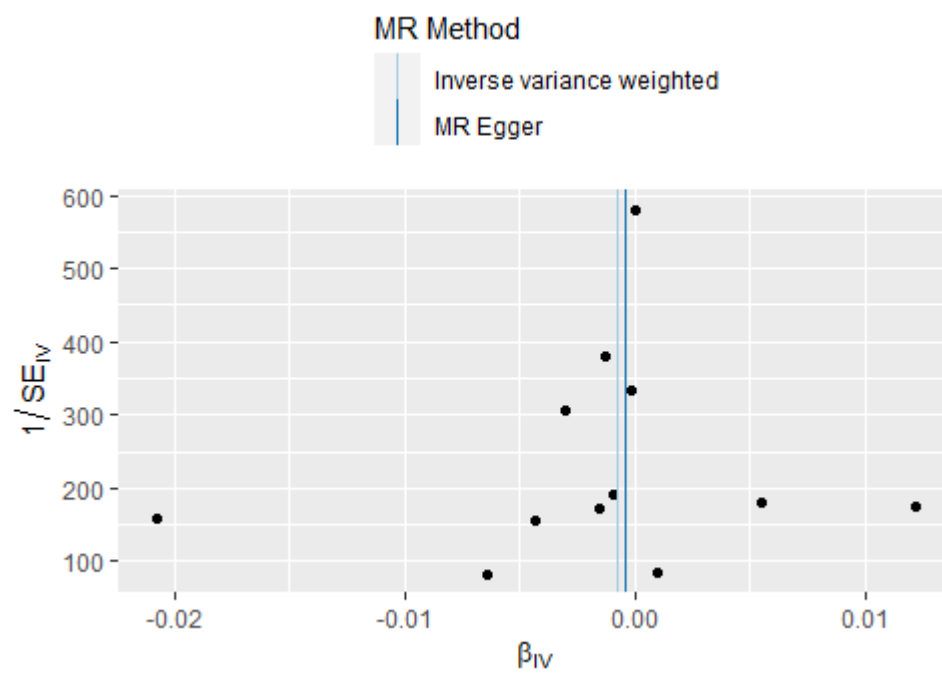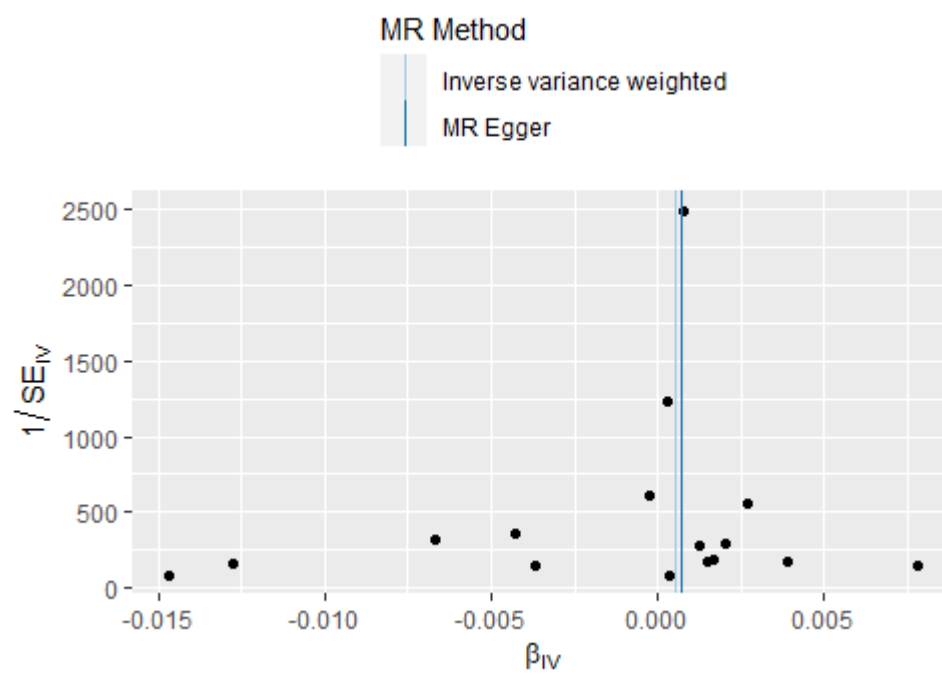

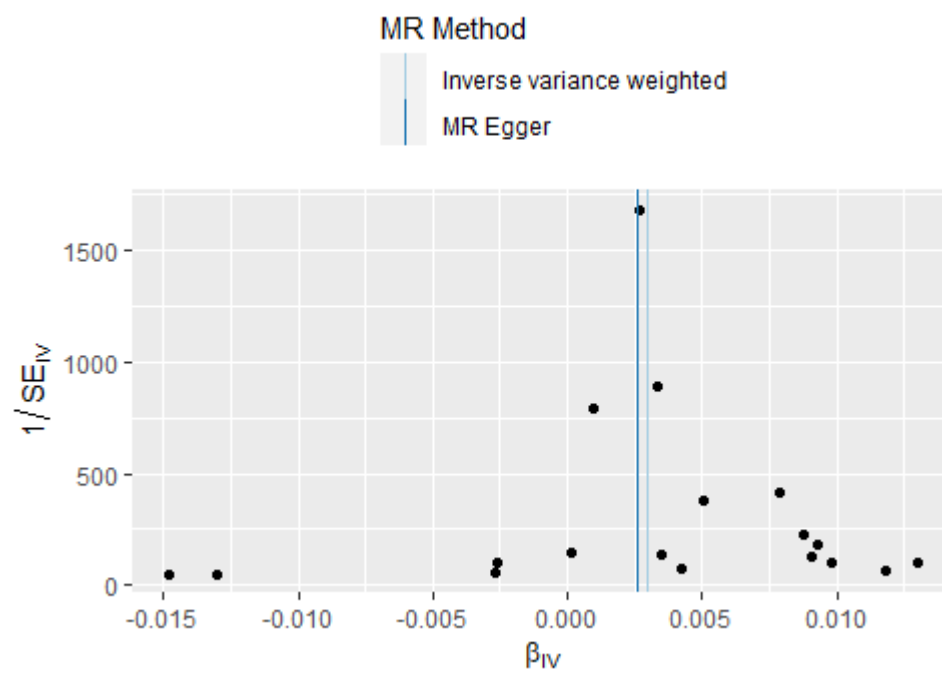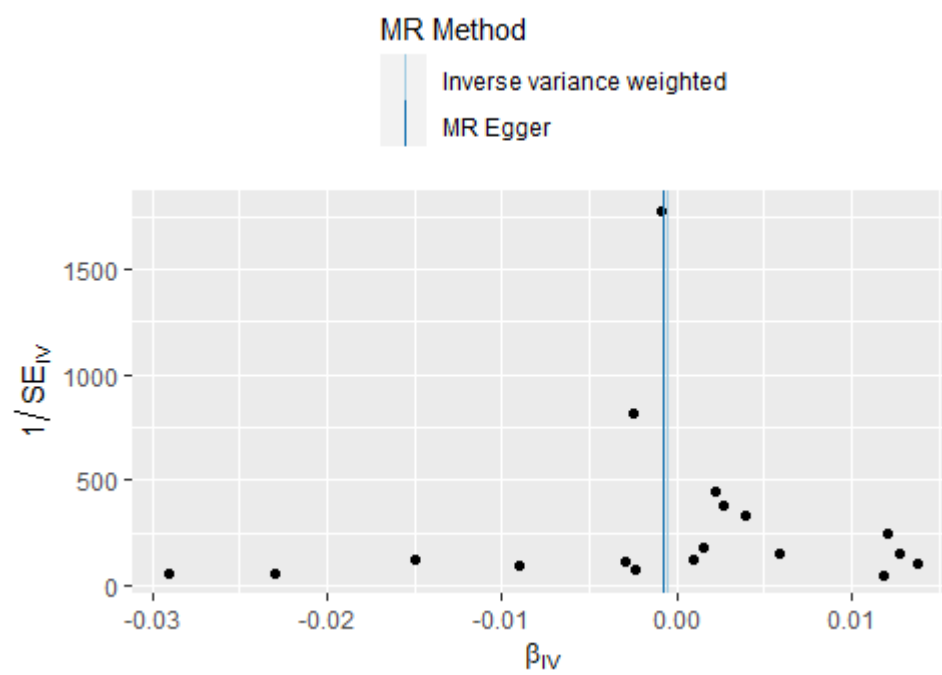

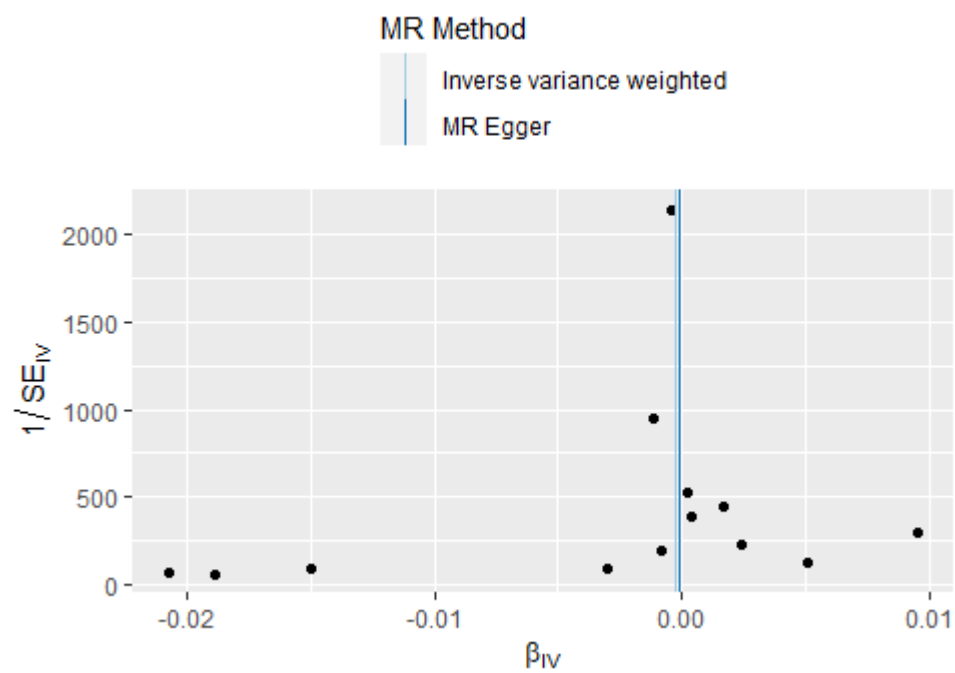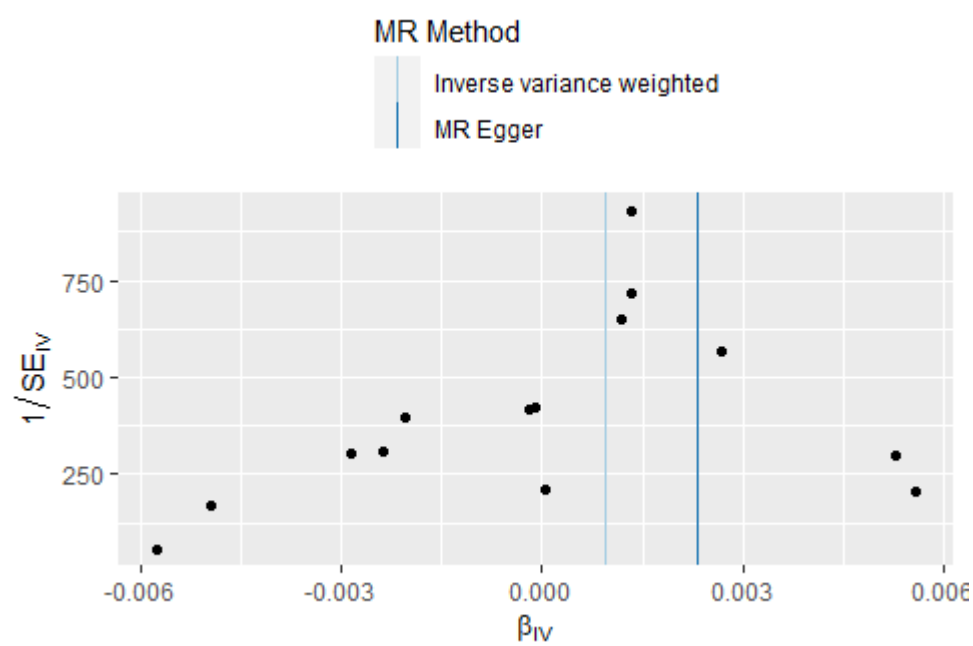

Supplement: Supplementary file 3 — Additional file 3: Figure S3. The funnel plots of Lp(a) on CVD. [file 40001_2022_825_MOESM3_ESM.pdf]

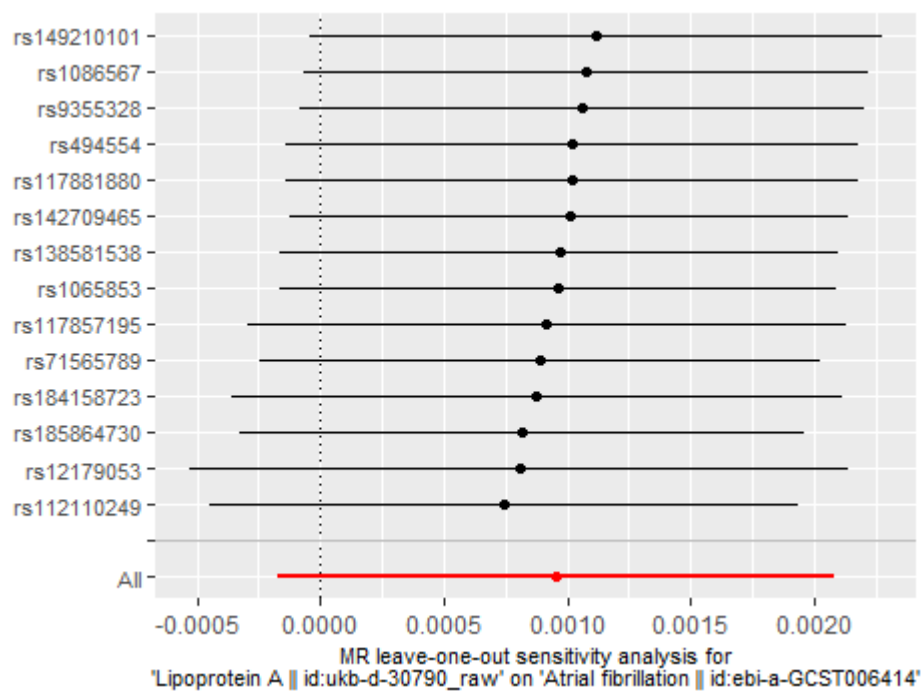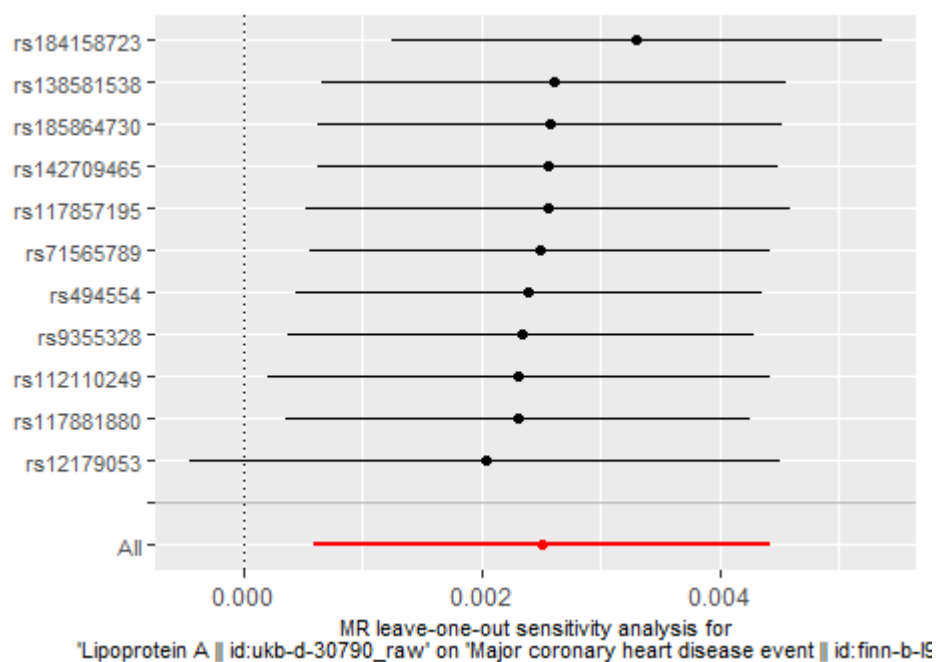

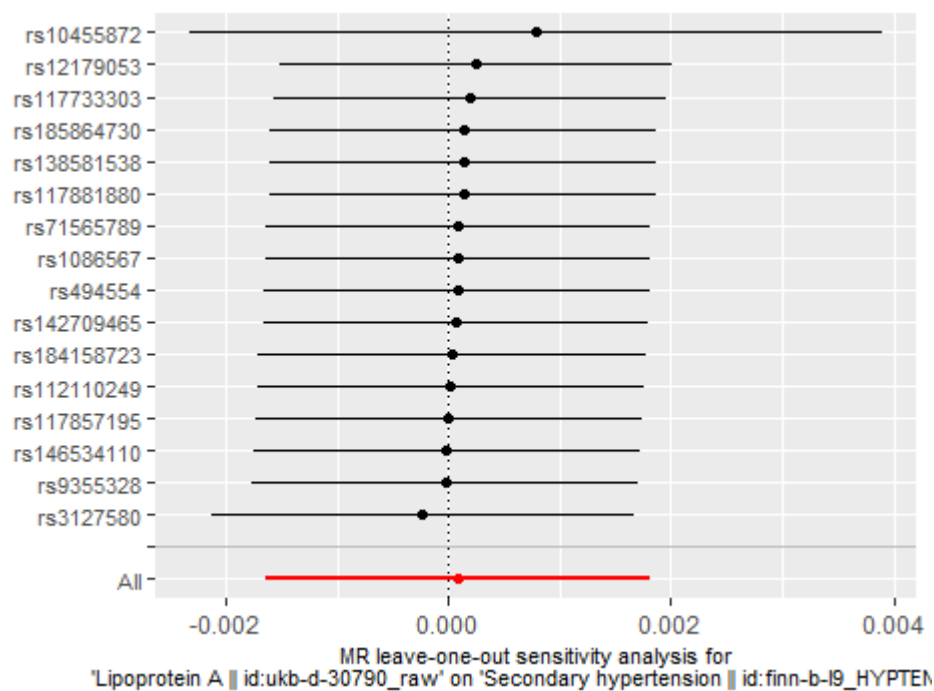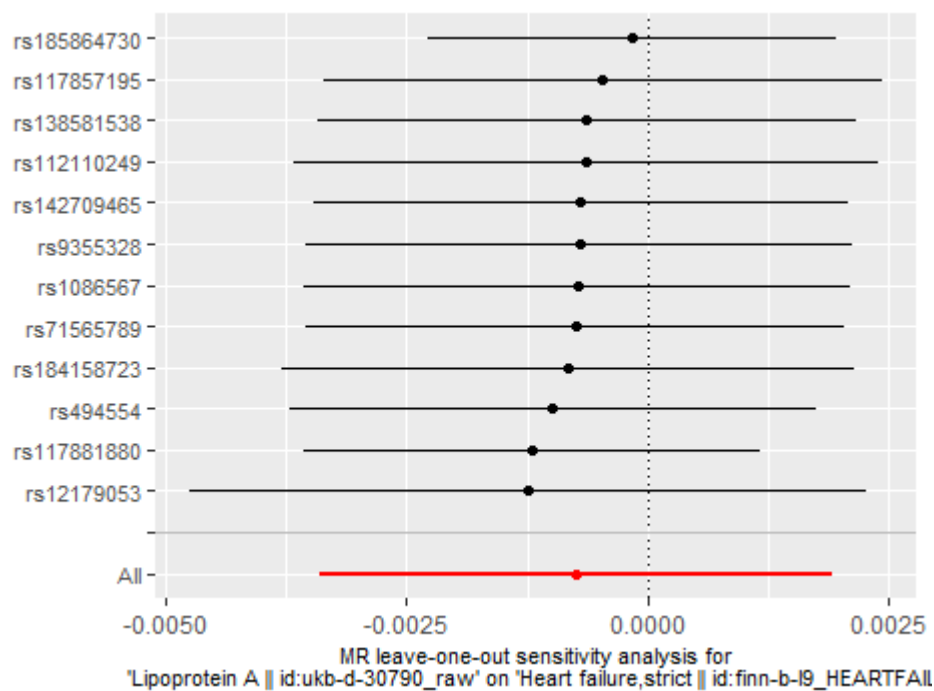

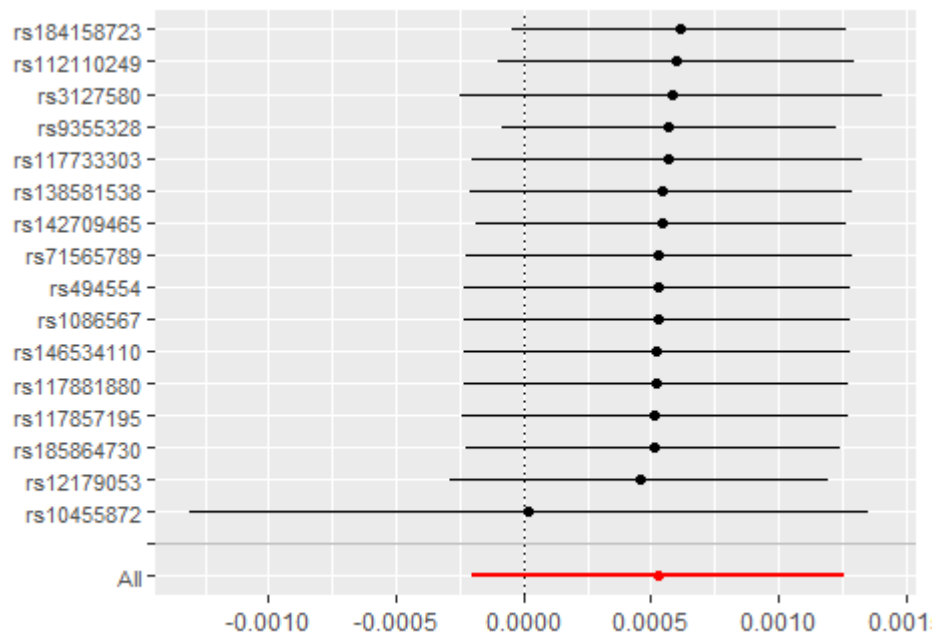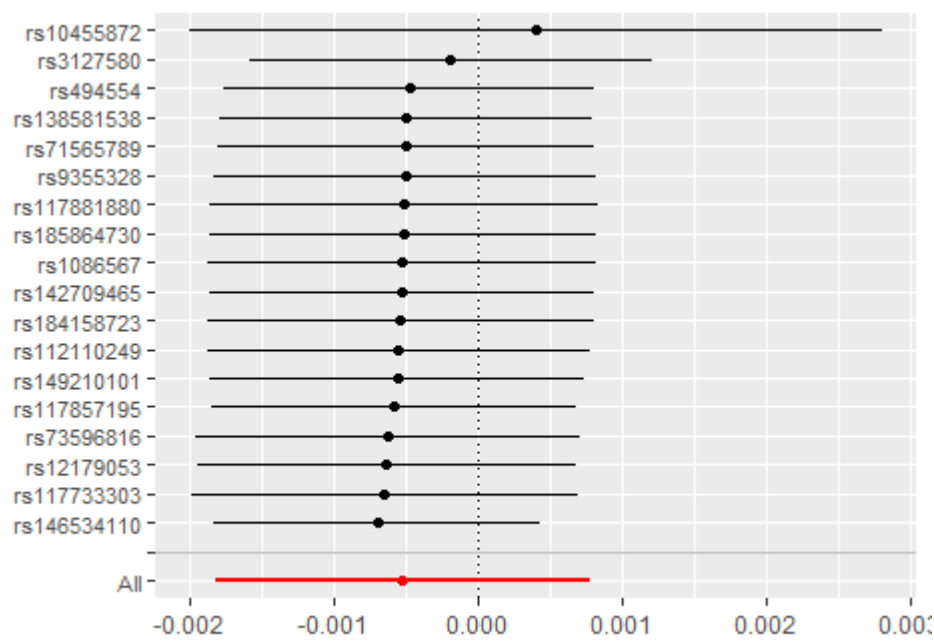

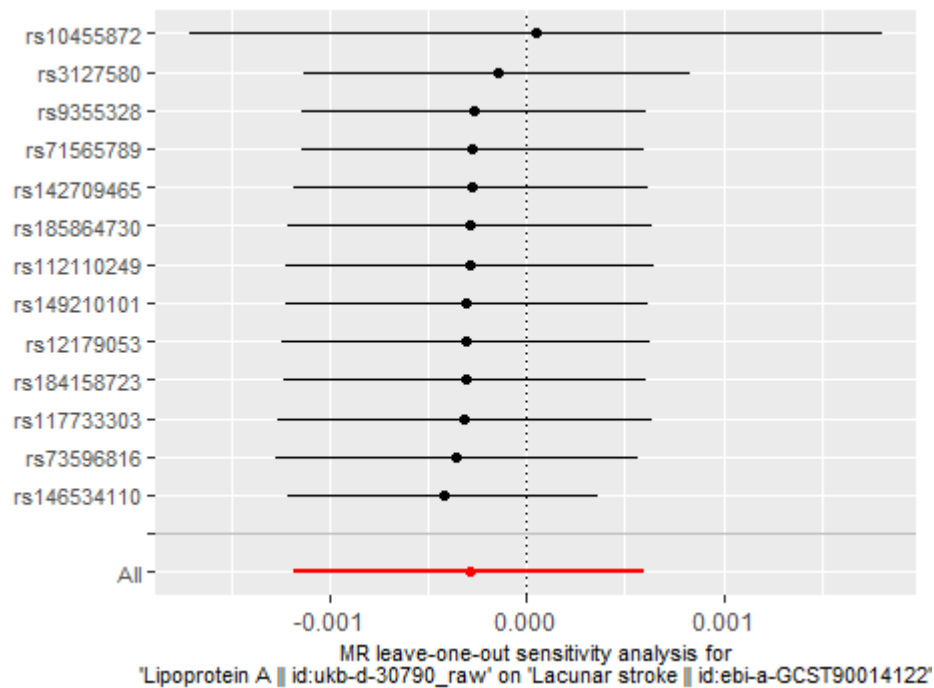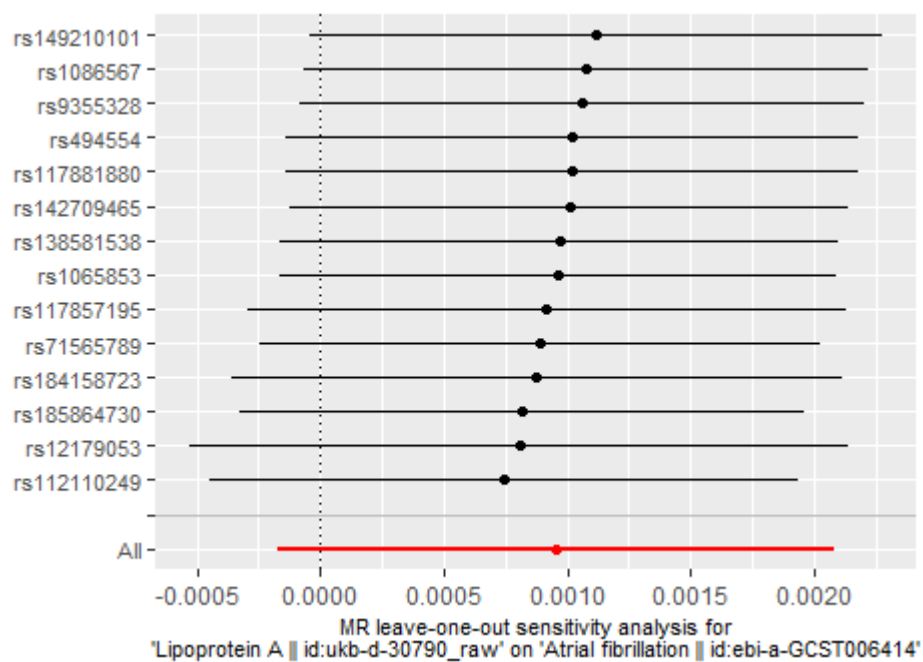

Supplement: Supplementary file 4 — Additional file 4: Figure S4. The leave-one-out method of Lp(a) on CVD (except AA and ISL). [file 40001_2022_825_MOESM4_ESM.pdf]
